# A Bayesian prediction model between a biomarker and the clinical endpoint for dichotomous variables

**DOI:** 10.1186/1745-6215-15-500

**Published:** 2014-12-20

**Authors:** Zhiwei Jiang, Yang Song, Qiong Shou, Jielai Xia, William Wang

**Affiliations:** Department of Health Statistics, School of Preventive Medicine, Fourth Military Medical University, No. 169 Changle West Road, Xi’an, Shaanxi China; Biostatistics and Research Decision Science, Merck Research Laboratory, Merck & Co., Inc., 2/F Building 22, Universal Business Park, No. 10 Jiuxianqiao Road, Beijing, China

**Keywords:** Biomarker, Clinical endpoint, Bayesian model, PPV, NPV

## Abstract

**Background:**

Early biomarkers are helpful for predicting clinical endpoints and for evaluating efficacy in clinical trials even if the biomarker cannot replace clinical outcome as a surrogate. The building and evaluation of an association model between biomarkers and clinical outcomes are two equally important concerns regarding the prediction of clinical outcome. This paper is to address both issues in a Bayesian framework.

**Methods:**

A Bayesian meta-analytic approach is proposed to build a prediction model between the biomarker and clinical endpoint for dichotomous variables. Compared with other Bayesian methods, the proposed model only requires trial-level summary data of historical trials in model building. By using extensive simulations, we evaluate the link function and the application condition of the proposed Bayesian model under scenario (i) equal positive predictive value (PPV) and negative predictive value (NPV) and (ii) higher NPV and lower PPV. In the simulations, the patient-level data is generated to evaluate the meta-analytic model. PPV and NPV are employed to describe the patient-level relationship between the biomarker and the clinical outcome. The minimum number of historical trials to be included in building the model is also considered.

**Results:**

It is seen from the simulations that the logit link function performs better than the odds and cloglog functions under both scenarios. PPV/NPV ≥0.5 for equal PPV and NPV, and PPV + NPV ≥1 for higher NPV and lower PPV are proposed in order to predict clinical outcome accurately and precisely when the proposed model is considered. Twenty historical trials are required to be included in model building when PPV and NPV are equal. For unequal PPV and NPV, the minimum number of historical trials for model building is proposed to be five. A hypothetical example shows an application of the proposed model in global drug development.

**Conclusions:**

The proposed Bayesian model is able to predict well the clinical endpoint from the observed biomarker data for dichotomous variables as long as the conditions are satisfied. It could be applied in drug development. But the practical problems in applications have to be studied in further research.

**Electronic supplementary material:**

The online version of this article (doi:10.1186/1745-6215-15-500) contains supplementary material, which is available to authorized users.

## Background

The biomarker employed as a surrogate to evaluate efficacy is preferred in clinical trials. It leads to reduced trial durations and costs [1, 2], improved compliance [3], better ethical satisfaction [4], *etcetera* For example, United States Food and Drug Administration (FDA) has enabled accelerated marketing approval based on a surrogate endpoint, rather than the primary endpoint, in life-threatening diseases [5, 6]. Controversy has surrounded the evaluation of a biomarker as a surrogate [7–20] since Prentice [7] formulated a statistical framework in the context of hypothesis testing. However, the evaluation of a biomarker covers various considerations [21–23]. The experience of the evaluation is limited and very few biomarkers are accepted as surrogate endpoints in practice [21]. On the other hand, as an intermediate endpoint, the biomarker is still helpful to predict the effect on clinical endpoint from the intervention even it cannot directly replace the true clinical endpoint as a surrogate. In drug development, the prediction of a true clinical endpoint from an early biomarker makes sense in early drug efficacy evaluation and decision making. The key point here is how to build and evaluate the prediction model between biomarker and clinical endpoint.

A meta-analytic approach was first considered by Fleming [11] and Hughes *et al*. [12] to validate a surrogate endpoint from a collection of previous trials. Some approaches modeled the association between a biomarker and clinical endpoint based on trial-level summary data in a frequentist framework [16–19]. But few have made such evaluations within the Bayesian framework. The main paper involving a Bayesian meta-analytic method is that of Daniels and Hughes [13], who considered a mixed effect model for the trial-level association of treatment effect on biomarker and clinical outcome. However, it required patient-level data to estimate the correlation between biomarker and clinical endpoint in the model building [24]. Both patient-level data and trial-level summary data were necessary for the method of Daniels and Hughes. Furthermore, Van Walraven *et al.* [24] implemented a Monte Carlo model and Wang *et al.* [25] employed classical Cox model to predict clinical outcomes from biomarkers.

The evaluation of the model between the biomarker and the clinical endpoint is another key concern. It is vital that the model is able to describe the association between the biomarker and the clinical outcome well and to predict the clinical endpoint from the observed biomarker accurately and precisely in a new trial. Buyse *et al.* [14] proposed the coefficient of determination  to evaluate the trial-level association and predictive ability. But the value of  is difficult to interpret [20], and it is difficult to clearly define which model is appropriate for prediction. On the other hand, the accessibility of trial-level summary data from historical trials is one of the advantages of meta-analytic approaches. But this approach also leads to information loss compared with the use of patient-level data. Therefore, simulation studies [17], which evaluate the model based on the simulative data from a specific assumption, ignore the effect of information loss from trial-level data and may over-evaluate the predictive ability of meta-analytic model. It is more objective and reasonable to evaluate the meta-analytic model based on the assumption of the patient-level data of previous trials in the simulations.

In this paper, we consider a Bayesian meta-analytic method for building a prediction model between the biomarker and the clinical endpoint when both endpoints are dichotomous, which is completely independent of patient-level data. It is used to predict rate ratio (*RR*) of the clinical endpoint from an early biomarker. In addition, we evaluate the proposed prediction model by using extensive simulations from clinical practical considerations. The patient-level data are simulated to evaluate the meta-analytic prediction model to avoid the assumption on trial-level data. Positive predictive value (PPV) and negative predictive value (NPV), which measure the patient-level association between biomarker and clinical endpoint for dichotomous variables, are employed in the simulations.

From the clinical point, a good biomarker is expected to have higher PPV and NPV to predict long-term clinical outcome (for example, in the early detection of a disease in clinical diagnosis, the early observed recurrence of a tumor in oncology study and so on). But a biomarker with higher NPV and lower PPV is also common in medical studies. For example, persistent infection of HPV (human papillomavirus) is considered to be a potential biomarker of high grade cervical disease and eventually cervical cancer. Because though HPV persistent infection case does not always progress to high grade cervical disease, the one who is not infected with HPV persistently has only a small probability of getting the disease from the viewpoint of medical mechanisms. In HPV vaccine clinical trials, a persistent infection of HPV, as a biomarker that has higher NPV and lower PPV, is able to help predict vaccine efficacy. Therefore, both scenarios, (i) equal PPV and NPV and (ii) higher NPV and lower PPV, are considered in the evaluation of the proposed model in the simulations.

The proposed prediction model between biomarker and clinical outcome in this article is built in a Bayesian framework. It is different from the frequentist meta-analytic approaches [14–19, 26]. It intuitively describes the association between biomarker and clinical endpoint and is easy to be implemented by using Markov Chain Monte Carlo (MCMC) techniques. But compared with other Bayesian methods, the proposed model has its own features. The Bayesian mixed model proposed by Daniels and Hughes [13] is not a complete meta-analytic method and the advantage of the meta-analytic approach cannot be shown. But the proposed Bayesian model has no such restriction. Furthermore, the method of Daniels and Hughes ignores the variability of within-trial treatment effect [10], which is included in the proposed Bayesian model.

## Methods

### Bayesian model for prediction

Consider *N* randomized trials of size *n*_*i*_(*i* = 1, 2, …, *N*). Equal sample size is assumed in the treatment and control group in each trial. In the *i*-th trial, *B*_*Ti*_ and *B*_*Ci*_ biomarker responses are observed in the treatment and control group, respectively. *X*_*Ti*_ and *X*_*Ci*_ subjects respond to the clinical outcome in the two groups. Let *φ*_*Bi*_ be the proportion of biomarker responses in the treatment group in the *i*-th trial. It is estimated by . Correspondingly,  is the estimate of *φ*_*Xi*_, which denotes the proportion of clinical outcome responses in the treatment group. Assuming the association between the biomarker and the clinical endpoint is equal across *N* trials irrespective of the intervention, a generalized linear model
1

is proposed to describe the relationship. In the model, *g*(⋅) is a link function. The biomarker and clinical endpoint are transformed by using the link function because they are both dichotomous. In this paper, three link functions, the odds function:
2

the logit function:
3

and the cloglog function:
4

are considered and will be further compared in the simulations.

Given *B*_*i*_, *B*_*Ti*_, *X*_*i*_ and *X*_*Ti*_, *φ*_*Bi*_ and *φ*_*Xi*_ follow the distributions of
5

and
6

in which *B*_*i*_ = *B*_*Ti*_ + *B*_*Ci*_ and *X*_*i*_ = *X*_*Ti*_ + *X*_*Ci*_.

In the Bayesian model, we consider a uniform prior for the coefficient *β* and a normal prior for the intercept *β*_0_ with mean zero and variance *σ*^2^. The *φ*_*Bi*_ takes a prior distribution of *Beta*(*a*_*Bi*_, *b*_*Bi*_). The non-informative priors are considered for all three parameters *β*, *β*_0_ and *φ*_*Bi*_. The posterior distributions of the three parameters depend on the link function in the model. Given *B*_*i*_, *B*_*Ti*_, *X*_*i*_, *X*_*Ti*_ and a specific link function, the estimates of posterior distributions for *β*, *β*_0_ and *φ*_*Bi*_ are calculated by using the MCMC method based on the formulas (1), (5) and (6). The uncertainty on within-trial treatment effect is considered by incorporating formula (5) and (6) in the model.

**Figure 1:**
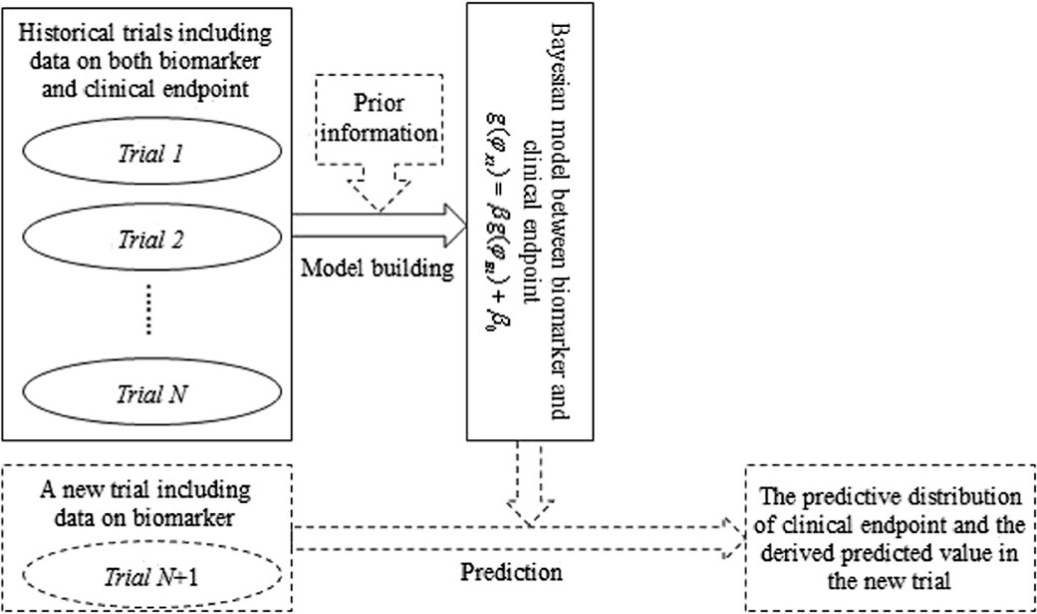
**The diagram of Bayesian model building and prediction.**

For a new trial *j*, the rate ratio of the treatment and control group on the clinical endpoint
7

is employed to evaluate the efficacy of the intervention. An equal association between the biomarker and the clinical endpoint across the new trial and historical trials is assumed here. It means that the biomarker in the new trial captures the same treatment effect as the one in the historical trials. Based on the Bayesian model built from *N* historical trials, the MCMC estimation of predictive distribution for *φ*_*Xj*_ is obtained when *φ*_*Bj*_ is given. Correspondingly, the predictive distribution of *RR*_*j*_ is derived from formula (7). We take the median of the predictive distribution as the point estimate of *RR*_*j*_ prediction and construct the 95% credible interval (CI) with 2.5% and 97.5% percentiles. The flow chart of Bayesian model building and prediction is depicted in Figure 1.

The predictive ability of the proposed model is related to the association strength between the biomarker and the clinical outcome. PPV and NPV are generally employed to measure the association from the patient level. These directly affect the predictive ability of the proposed Bayesian model. When one uses the proposed approach to build the Bayesian model and to predict the clinical outcome of the new trial, the first thing is to evaluate the strength of the association between the biomarker and the clinical endpoint, which is measured by PPV and NPV. Therefore, a simulation study is conducted to explore the application condition of the proposed method.

### Simulation study

A simulation study is employed to (a) compare the predictive ability of different link functions (the odds, logit and cloglog functions) in the proposed Bayesian model when PPV and NPV vary; (b) explore the effect of the association strength between biomarker and clinical endpoint, which is measured by PPV and NPV, on the predictive ability of the model; and (c) discuss the number of historical trials to be included in model building for a good clinical prediction in the new trial. As we have mentioned above, the biomarkers with (i) equal PPV and NPV and (ii) higher NPV and lower PPV are both common in medical studies. From the practical perspective, both scenarios are considered in the simulations. All simulations are repeated for 5,000 times and performed by using R package for data generation and calculation and OpenBUGS for Bayesian model fitting.

#### Data generation process

In *N* historical randomized trials, it is assumed that the biomarker response rate of control group is 0.3, and that 400 patients complete the trial for simplicity. The sample size ratio of treatment and control group is 1:1.

Let *π*_*BTi*_ and *π*_*BCi*_ be the biomarker response rate of the treatment and control group in the *i*-th trial. Then *φ*_*Bi*_ = *π*_*BTi*_/(*π*_*BTi*_ + *π*_*BCi*_). when equal sample size in the two groups. Because 0 < *π*_*BTi*_ <1 and *φ*_*Bi*_ has to be 0 < *φ*_*Bi*_ <0.76 when *π*_*BCi*_ is specified as 0.3. Therefore, we consider *φ*_*Bi*_ comes from the uniform distribution *U*(0, 0.76) and the biomarker response rate of the treatment group *π*_*BTi*_ is derived when *π*_*BCi*_ = 0.3 in the simulations.

Let *D*_*pi*_ be the biomarker response identifier of the *p*-th patient in the *i*-th trial and  Here, *D*_*pi*_ = 1 when the biomarker responds; otherwise, *D*_*pi*_ = 0. It is randomly generated from *D*_*pi*_ ~ *Bernoulli*(*π*_*BTi*_) for the treatment group and *D*_*pi*_ ~ *Bernoulli*(*π*_*BCi*_) for the control group.

Let *ρ* be the concordance index between *C*_*pi*_ and *D*_*pi*_, where *C*_*pi*_ denotes the clinical response identifier of the *p*-th patient in the *i*-th trial and *ρ* is from the distribution of *Bernoulli*(PPV) for *D*_*pi*_ = 1 and *Bernoulli*(NPV) for *D*_*pi*_ = 0. The patient-level data of clinical endpoint *C*_*pi*_ could be derived by using
8

Finally, the summary statistics of each historical trial, *B*_*i*_, *B*_*Ti*,_*X*_*i*_ and *X*_*Ti*_, are derived from the patient-level data and employed to build the Bayesian model by using formulas (1), (5) and (6).

#### The calculation of true values

For a new trial *j* with sample size of 400, given PPV, NPV and *π*_*BTj*_, the true value of the clinical response rate of the treatment group is calculated by using
9

Equally, the true value of clinical response rate of control group *π*_*XCj*_ is calculated and the true value of *RR*_*j*_ is derived from *RR*_*j*_ = *π*_*XTj*_/*π*_*XCj*_.

#### Measures of predictive ability

The accuracy and robustness of *RR* prediction is vital to measure the predictive ability of the proposed Bayesian model. But the traditional measures, for example, bias, root mean square error (RMSE), *etcetera*, cannot be applied here because the endpoint is binary. The log transformation is considered to adjust the non-normality of the binary endpoint. The modified bias and modified RMSE are proposed to measure the accuracy of the *RR* prediction. For the new trial *j*, when *K* predicted values of *RR*_*j*_ are obtained, they are calculated by using
10

and
11

in which  denotes the true value of *RR*_*j*_ and  is the *k*-th predicted value of *RR*_*j*_. Similarly, modified RMSE includes the effect of bias direction to estimate prediction error compared with modified bias. The *RR* prediction is more accurate when the modified bias and the modified RMSE approach to 0.

To measure the precision of the *RR* prediction, the average width of the 95% CIs is calculated by using
12

where  and  are the upper and lower bound of 95% CI of the *k*-th *RR*_*j*_ prediction respectively. When the average width of 95% CIs approaches to 1, the better the precision of *RR* prediction is.

## Results

### Simulation I: comparison of Bayesian model with different link functions and positive predictive values/negative predictive values

Three link functions ( the odds, logit and cloglog functions) are compared in this section. The *φ*_*Bj*_ is assumed to be 0.1, 0.3, and 0.5. Ten historical trials are generated randomly from patient level and their trial-level summary data are included for Bayesian model building. Modified bias and modified RMSE are calculated to measure the accuracy of *RR* prediction, and average width of 95% CIs is estimated to evaluate the precision. Both scenarios, (i) equal PPV and NPV and (ii) higher NPV and lower PPV, are considered here.

#### Scenario I: equal PPV and NPV

**Figure 2:**
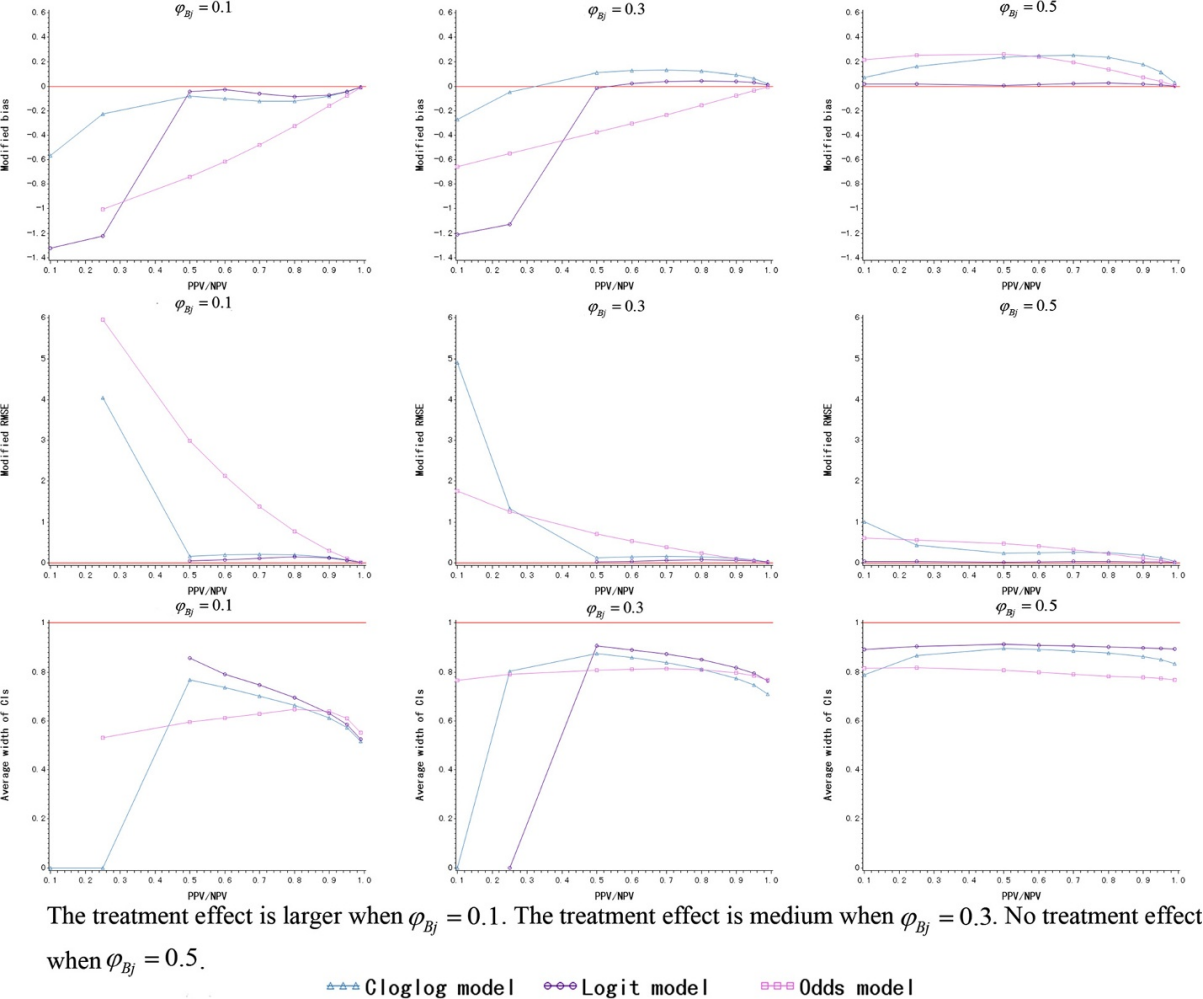
**The simulation results of Bayesian model for different link functions and** ***φ***_***Bj***_ **(** ***N*** **= 10, equal positive predictive value and negative predictive value).** *(PPV: positive predictive value; NPV: negative predictive value)*

In the simulations, PPV/NPV is assumed to take the values of 0.1, 0.25, 0.5, 0.6, 0.7, 0.8, 0.9, 0.95 and 0.99. Figure 2 depicts the change of modified bias, modified RMSE and average width of 95% CIs for different *φ*_*Bj*_ and link functions. No matter which link function is employed, the modified bias and the modified RMSE are both worse when PPV/NPV <0.5. The modified RMSE fails to be estimated for the logit link function when PPV/NPV <0.5 and *φ*_*Bj*_ ≤0.3 because of the poor prediction. The other two link functions have the same problems when PPV/NPV = 0.1 and *φ*_*Bj*_ = 0.1. On the other hand, the average width of the 95% CIs is not good when PPV/NPV <0.5, especially when *φ*_*Bj*_ <0.5. Therefore, PPV/NPV ≥0.5 is an indispensable condition of the proposed Bayesian model for *RR* prediction under the assumption of equal NPV and PPV.

Among the three link functions, both modified bias and modified RMSE of the logit function are the closest to zero when PPV/NPV ≥0.5, and the cloglog link function is better than the odds function. For the logit link function, modified bias and modified RMSE have a little inflation when PPV/NPV = 0.8 and approach to zero when PPV/NPV continues increasing. The accuracy of the prediction remains good, however, if *φ*_*Bj*_ varies when PPV/NPV ≥0.5 and logit link function is employed. Regarding the precision of the *RR* prediction, the logit link function has the narrowest average width of the 95% CIs, which increases and approaches to the other two link functions when PPV/NPV increases. As *φ*_*Bj*_ increases, the treatment effect becomes smaller and the average width of 95% CIs of the logit link function decreases. The logit link function is considered as the first choice for building the Bayesian model from the perspective of the accuracy and precision of *RR* prediction. The detailed simulation results are presented in Additional file 1: Table S1.

#### Scenario II: unequal positive predictive value and negative predictive value

**Figure 3:**
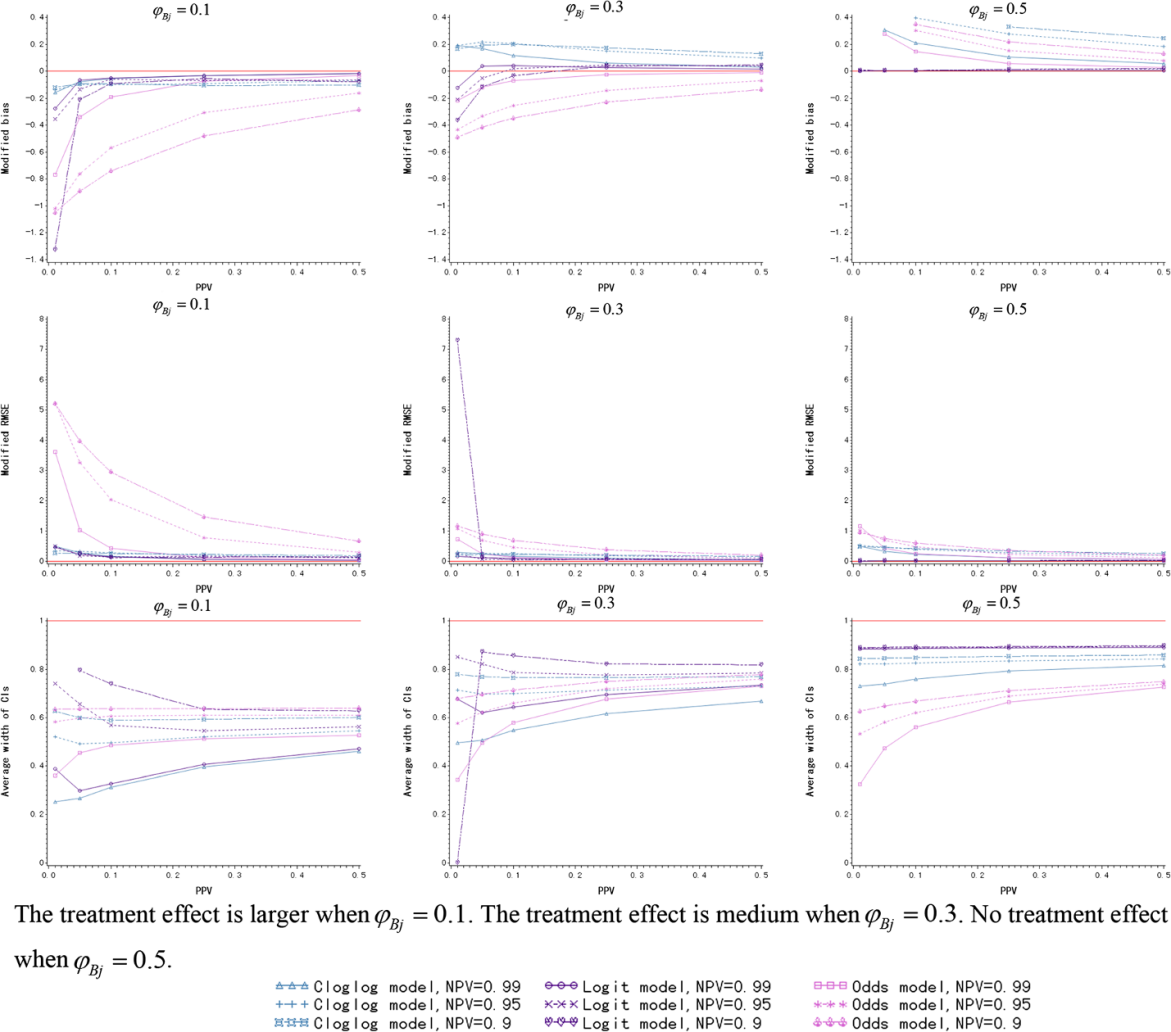
**The simulation results of Bayesian model for different link functions and** ***φ***_***Bj***_ **(** ***N*** **= 10, higher negative predictive value and lower positive predictive value).** *(PPV: positive predictive value; NPV: negative predictive value)*

In this scenario, NPV is considered to be 0.99, 0.95 and 0.9, and PPV is not larger than 0.5. The values of PPV and NPV are listed in Additional file 1: Table S2. As is depicted in Figure 3, the logit and cloglog link function have the better prediction accuracy on modified bias and modified RMSE when PPV + NPV ≥1. But the odds link function leads to an inaccurate prediction, especially when *φ*_*Bj*_ = 0.1. As *φ*_*Bj*_ increases, the modified bias of the cloglog link function is rising and deviates from zero when *φ*_*Bj*_ ≥0.3 though the modified RMSE of cloglog one still looks fine. However, the modified bias of the logit link function always fluctuates around zero regardless of how *φ*_*Bj*_ varies.

On the other hand, the average width of the 95% CIs of the logit link function is a little narrower than cloglog one when PPV/NPV remains the same and PPV + NPV ≥1. Considering both the accuracy and precision of the prediction, the logit link function is also the optimal one to build the Bayesian model when PPV + NPV ≥1 under the assumption of higher NPV and lower PPV. Furthermore, the average width of the 95% CIs of the logit link function is a little larger when *φ*_*Bj*_ = 0.1 and becomes smaller when *φ*_*Bj*_ increases. When PPV is fixed, the logit function has the narrower average width of 95% CIs, as NPV is smaller.

### Simulation II: the number of historical trials to be included for model building

The number of historical trials to be included for model building is a key issue to meta-analytic approaches. In this section, 5, 10, 20, 30 and 50 historical trials are assumed to build the Bayesian prediction model, and their predictive abilities are compared under (i) equal PPV and NPV and (ii) higher NPV and lower PPV. Based on the simulation results of the last section, the logit link function is employed to build the Bayesian model here. The observed *φ*_*Bj*_ of the new trial is still assumed to be 0.1, 0.3, and 0.5.

#### Scenario I: equal positive predictive value and negative predictive value

**Figure 4:**
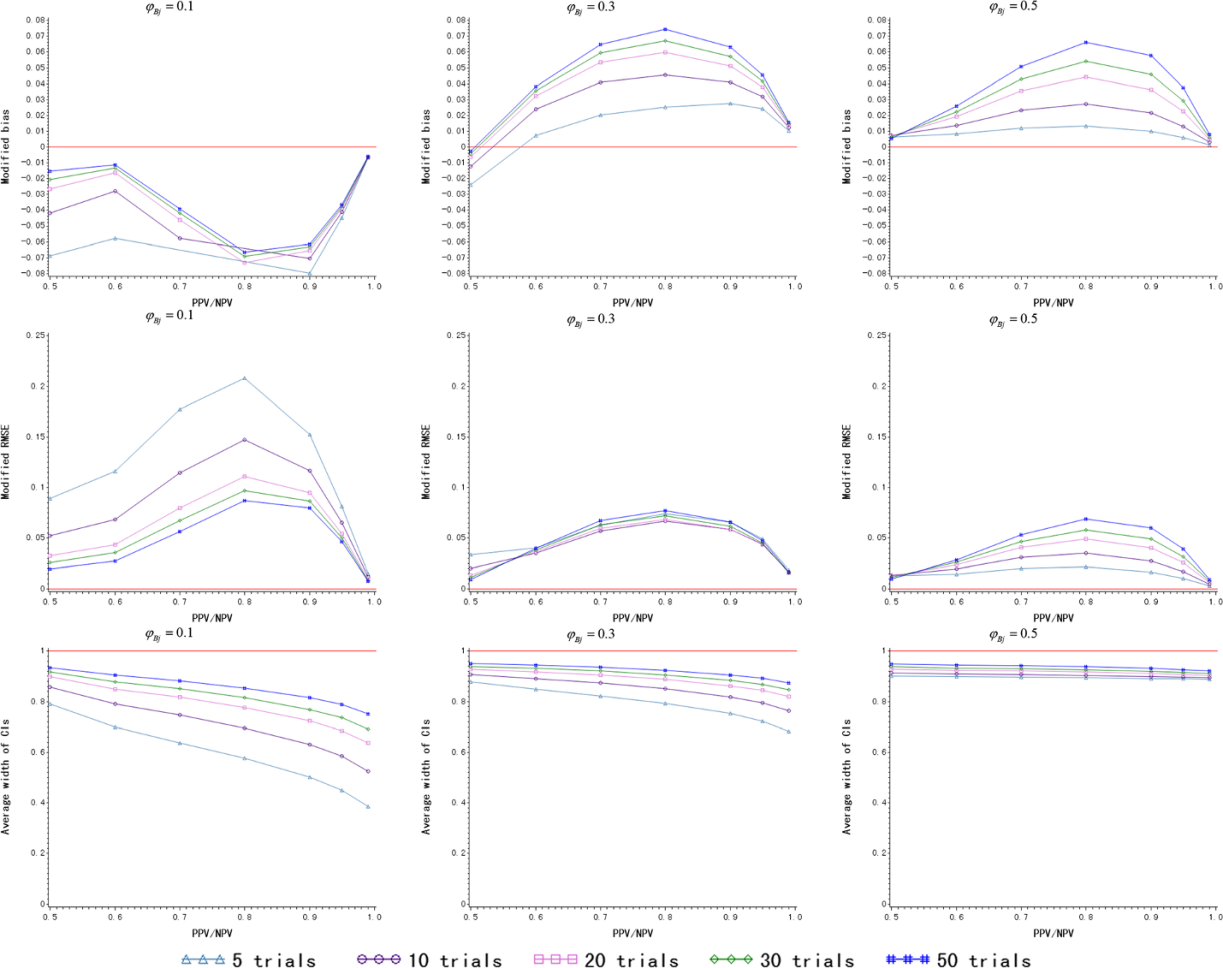
**The simulation results of Bayesian model for various** ***N*** **and** ***φ***_***Bj***_ **given logit link function (equal positive predictive value and negative predictive value).** *(PPV: positive predictive value; NPV: negative predictive value)*

Based on the results of the last section, PPV/NPV ≥0.5 is considered here. The values of PPV/NPV are listed in Additional file 1: Table S3. As is seen in Figure 4, the prediction is a little underestimated when *φ*_*Bj*_ = 0.1. The modified bias increases and even deviates from zero positively with the rise of *φ*_*Bj*_. It varies within (-0.08,0.08) regardless of how *φ*_*Bj*_ changes. The modified RMSE is the smallest when 50 trials are included in model building for *φ*_*Bj*_ = 0.1. But as *φ*_*Bj*_ increases, the larger *N* does not always bring an accurate prediction. For example, the model built from five trials has the smallest modified RMSE when *φ*_*Bj*_ = 0.5. It is perhaps because there is no treatment effect when *φ*_*Bj*_ = 0.5, and more historical trials include larger variations in the model, which reduces the accuracy of the prediction. Regarding the precision of *RR* prediction, the average width of the 95% CIs is smaller when *N* is larger and *φ*_*Bj*_ increases. The detailed simulation results are presented in Additional file 1: Table S3.

When *N* = 20, the maximum modified RMSE is only about 0.1, and the average width of the 95% CIs is also larger than 0.6 even when *φ*_*Bj*_ = 0.1. Therefore, the minimum number of historical trials to be included for model building is proposed to be 20 for an accurate and precise prediction when PPV and NPV are equal.

#### Scenario II: unequal positive predictive value and negative predictive value

In the simulations, NPV is considered to be 0.99, 0.95 and 0.9 and PPV ≤0.5. As long as PPV + NPV ≥1 is satisfied, the accuracy of the prediction is good on both modified bias and modified RMSE. They both converge to zero when *N* and *φ*_*Bj*_ increase. The larger PPV + NPV is, the more accurate the prediction. Even when *N* = 5, the modified RMSE is smaller than 0.4 for *φ*_*Bj*_ = 0.1. Therefore, five historical trials are enough to build a model that has an accurate prediction.

**Figure 5:**
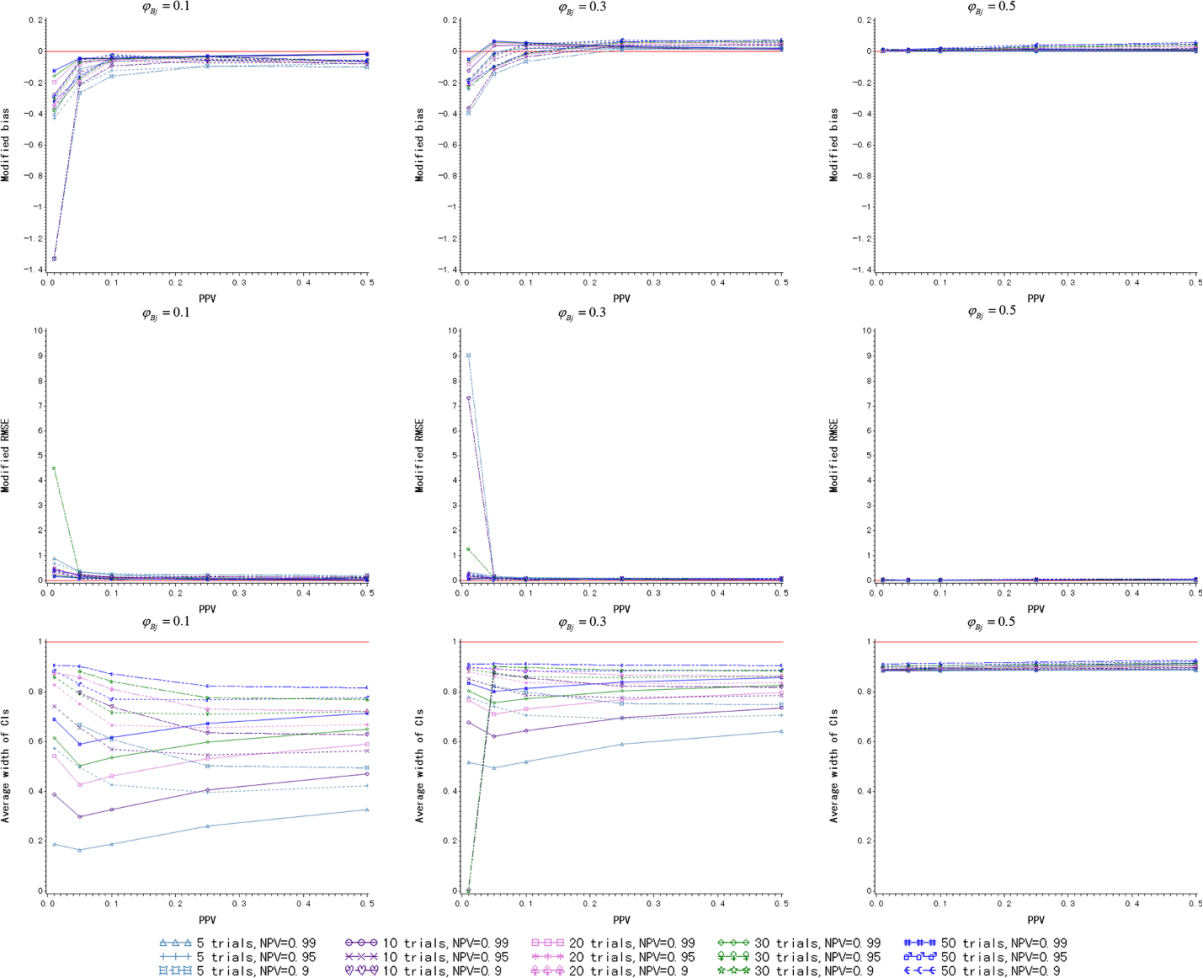
**The simulation results of the Bayesian model for various** ***N*** **and** ***φ***_***Bj***_ **given logit link function (higher negative predictive value and lower predictive value).** *(PPV: positive predictive value; NPV: negative predictive value)*

On the other hand, the larger *N* leads to the smaller average width of 95% CIs, which is shown in Figure 5. When PPV and *φ*_*Bj*_ is fixed, the average width of 95% CIs rises with the increase of NPV. When *N* = 5 and *φ*_*Bj*_ = 0.1, the minimum average width of 95% CIs is only about 0.2. The 95% CIs of the *RR* prediction is wide and the Bayesian model brings a conservative interval estimate. In conclusion, when a higher NPV and a lower PPV are considered, the Bayesian model built from five historical trials is enough for an accurate point estimate of the prediction, but leads to a conservative interval estimate. If a precise interval estimate of *RR* prediction is expected, *N* = 30 is proposed where the average width of 95% CIs is larger than 0.5 even when *φ*_*Bj*_ = 0.1.

### Hypothetical example

To evaluate the efficacy of a vaccine in clinical trial, the incidence rate of the disease of interest is usually observed. Vaccine efficacy (VE) is estimated by using VE = 1 - *π*_*T*_/*π*_*C*_ = 1 - *RR*, in which *π*_*T*_ and *π*_*C*_ denote the incidence rate of the disease in the vaccine and control group. However, the occurrence of disease requires too long a follow-up time. Virus infection is considered as an early biomarker of the disease. It has an NPV of 99.99% and PPV of 15% for predicting the occurrence of the disease. VE is just a transformation of *RR*. It is possible to predict vaccine efficacy earlier when the infection cases are observed by employing our proposed approach.

It was hypothesized that five global placebo-controlled vaccine trials with sample size ratio of 1:1 completed in the United States, Europe and Japan. The number of disease cases and infection cases in the vaccine and placebo group in each trial are listed in Table 1. Recently, a new placebo-controlled trial was conducted in China to evaluate VE in the Chinese population. A total of 3606 subjects in two groups of equal size were recruited. A total of 36 infection cases were observed, with 7 cases from the vaccine group. No disease was observed, and we wanted to evaluate vaccine efficacy earlier based on the infection cases.

**Table 1:** **Five hypothetical historical vaccine trials**

	***N***	Number of infection cases		Number of disease cases		Vaccine efficiency(95% CI) on disease
		Vaccine	Placebo	Vaccine	Placebo	
1	2954	2	213	0	23	100.0%(82.5%,100.0%)
2	3200	10	86	1	6	83.3%(-37.6%,99.6%)
3	529	2	45	0	2	100.0%(-431.4%,100%)
4	3674	27	130	3	8	62.7%(-55.5%,93.6%)
5	3452	24	296	0	30	100.0%(87.1%,100.0%)

CI: credible interval.

It is presumed that there is no ethnic difference in the association between infection and disease occurrence even if the vaccine efficacy is different between Chinese and other ethnic groups. The proposed approach can be applied to build the Bayesian model from historical vaccine trials and to predict VE from the observed infection cases in the Chinese population. A Bayesian model built from five historical vaccine trials in the United States, Europe and Japan could bring an accurate point estimate and a conservative interval estimate of VE prediction according to the simulation results in last section. Logit link function is employed in the Bayesian model. The VE on disease in the Chinese population is predicted as 81.84% with 95% CI of (53.98%,97.54%). Consequently, the vaccine is considered to have significant efficacy in the Chinese population because the lower boundary of the conservative 95% CI is still larger than zero.

## Discussion

The proposed Bayesian prediction model between a biomarker and the clinical outcome in this article is one of meta-analytic approaches. It focuses on the prediction of clinical outcome based on biomarker, not the replacement as a surrogate. According to Prentice criterion [7], the proposed Bayesian model requires that the association between the biomarker and the clinical endpoint be equal across the two groups. Compared with the Daniels and Hughes method, the proposed Bayesian model is a complete meta-analytic approach, which only requires the accessible trial-level summary data of previous trials, and no patient-level data is necessary. Nikolakopoulos *et al.* [27] also employed a Bayesian method to predict clinical endpoint based on observed biomarker for phase II trial decision making. But there are important differences between the two approaches. First, the model between a biomarker and the clinical endpoint must be known for clinical outcome prediction, and how to build the model from previous studies was not considered in [27]. But it is part of our work. Both model building from historical trials and model prediction for a new trial are considered here. Second, as a meta-analytic approach, our proposed model only involves trial-level summary data of historical studies for model building and predicts clinical endpoint with trial-level summary biomarker data for the new trial. The patient-level data of the new trial is not necessary for the prediction. PPV and NPV are not involved in model building and model prediction, but are needed for model evaluation. Third, the proposed prediction model is also different. Here, we propose a generalized linear model to describe the association between the biomarker and the clinical endpoint. We only consider the prior information on the model parameter *β*, *β*_0_ and *φ*_*Bi*_ for model building, but not place prior information on the clinical endpoint in the prediction. Finally, the scenario of unequal PPV and NPV between the biomarker and the clinical endpoint, which is common in vaccine clinical trials, is discussed in this article. On the other hand, the proposed Bayesian model describes the relationship between biomarker and clinical endpoint regardless of treatment effect. The historical trials for model building do not have to contain the same treatment and control group. The observational studies are also available for model building as long as both biomarker and clinical endpoint are collected in the studies. It further increases the availability of historical studies and makes it easy to be applied in practical.

In the proposed Bayesian model, *φ*_*Bi*_ and *φ*_*Xi*_ are employed to measure the treatment difference of biomarker and clinical outcome between the two groups and connected with formula (1). That is because this paper is to predict the rate ratio of clinical outcome for binary endpoint. *φ*_*Bi*_ and *φ*_*Xi*_, which are usually considered in vaccine clinical trials, have a direct connection with *RR* that is shown in formula (7) and does not depend on trial size. The association between the biomarker and clinical endpoint could be well described in the model in virtue of *φ*_*Bi*_ and *φ*_*Xi*_. When unequal sample size in the two groups is considered, *φ*_*Bi*_ is still calculated by *φ*_*Bi*_ = *π*_*BTi*_/(*π*_*BTi*_ + *π*_*BCi*_), but estimated by  where *R* is the sample size ratio of treatment and control group. Equally, *φ*_*Xi*_ is estimated by . Therefore, the proposed Bayesian model is still applicable for unequal sample size in two groups.

Though multiple meta-analytic models between biomarker and clinical outcome were proposed, few of them evaluated the model in a broad sense and explicitly describe the application guidance. The direct assumption on trial-level data in Baker’s simulation study [17] possibly influences the evaluation. The association model may be over-evaluated because the information loss from the trial-level data was ignored. , introduced by Buyse *et al.* [14], is difficult to interpret, and it is difficult to define the application condition clearly. To overcome both problems, we make assumption on the patient-level data of historical trials and perform the simulations to evaluate the meta-analytic model.

Both scenarios, (i) equal PPV and NPV and (ii) higher NPV and lower PPV, are considered in the simulations from the practical perspective. In clinical diagnosis, higher PPV and NPV are usually required for a biomarker to detect the potential disease earlier [27, 28]. But the biomarker with a higher NPV and lower PPV is also common in vaccine studies. According to the simulation results, the proposed Bayesian model leads to a good prediction of clinical *RR* based on a biomarker when both PPV and NPV are larger than 0.5. For higher NPV and lower PPV, the model makes sense of the prediction when PPV + NPV ≥1. In an actual trial, the exact values of NPV and PPV between biomarker and clinical outcome are generally unknown. But it is possible to estimate PPV and NPV within a range by clinicians from the medical mechanism and clinical experiences and evaluate if the model is applicable. Furthermore, the logit link function is better than other functions in model building from the point of accuracy and precision of *RR* prediction. But for a specific trial, an extensive simulation for the trial is recommended to choose the optimal link function for model building to satisfy the demand of the trial.

Regarding the minimum number of historical trials to be included in model building, it is advised that 20 historical trials be enough to build a model that predicts clinical *RR* accurately and precisely for equal PPV and NPV. When higher NPV and lower PPV is considered, the model built from five historical trials is able to lead to an accurate point estimate of the prediction, but a conservative interval estimate. If a more precise prediction is demanded, a larger *N* is required. However, if too low biomarker and clinical response rate is expected in all historical trials, more historical trials are proposed to be included in model building in order to describe the association between biomarker and clinical outcome accurately and well predict the clinical outcome from biomarker in the new trial. A simulation is also proposed to evaluate the minimum number of historical trials for a specific trial.

The proposed Bayesian model for clinical outcome prediction based on biomarker in this paper is not only for binary endpoint, but also continuous variables as long as a suitable link function is given. The proposed method has extensive potential applications in drug development. The example shows an application in global drug development. As long as an equal association between the biomarker and the clinical endpoint across different ethnic groups is considered from the medical point, it is possible to ‘bridge’ the relationship from historical global studies to a new regional trial in virtue of the proposed Bayesian model and then predict clinical endpoint in the new region. It is different from the traditional bridging study, which bridges the treatment effect from historical trials, and equally enhances the efficiency of regional trial in another way. On the other hand, the early prediction of clinical outcome with the help of the Bayesian model built from the historical studies is able to help with making a go/no-go decision in new drug development. However, a few practical problems, for example, sample size estimation of regional trial involving biomarker, go/no-go decision rule based on biomarker, *etcetera*, will be encountered in the application of the proposed Bayesian model. They are not in the scope of this article and will be discussed in further studies.

## Conclusions

A Bayesian prediction model between a biomarker and the clinical outcome is proposed in this paper. It is a complete meta-analytic approach and only requires trial-level data in model building. It is able to predict well the clinical outcome from an observed biomarker when PPV/NPV ≥0.5 for equal PPV and NPV and when PPV + NPV ≥1 for higher NPV and lower PPV. The Logit link function is preferred in both scenarios. The minimum number of historical trials to be included in model building is proposed to be 20 when PPV and NPV are considered to be equal. For higher NPV and lower PPV, the Bayesian model from five historical trials could lead to an accurate point estimate, but conservative interval estimate of the prediction. The proposed model has potential applications in decision making of new drug development and global-regional drug development program. But the practical problems have to be discussed in further studies.

## Electronic supplementary material

Additional file 1: Table S1: The simulation results of the Bayesian model for different link functions and *φ*_*Bj*_ under the assumption of equal PPV and NPV (*N* = 10). **Table S2.** The simulation results of the Bayesian model for the different link functions and *φ*_*Bj*_ under the assumption of higher negative predictive value (NPV) and lower positive predictive value (PPV) (*N* = 10). **Table S3.** The simulation results of the Bayesian model for the various *N* and *φ*_*Bj*_ given logit link function and equal PPV and NPV. **Table S4.** The simulation results of the Bayesian model for the various *N* and *φ*_*Bj*_ given logit link function and equal higher NPV and lower PPV. **Table S5.** Table of notations in the article. (DOC 887 KB)

## Abbreviations

NPV: negative predictive value
PPV: positive predictive value
RMSE: root mean square error
RR: rate ratio
VE: vaccine efficacy
HPV: human papillomavirus
MCMC: Markov Chain Monte Carlo
CI: credible interval
RMSE: root mean square error.
