# Supplementary material for: A Bayesian prediction model between a biomarker and the clinical endpoint for dichotomous variables
Source: Trials. 2014 Dec 20;15:500. doi: 10.1186/1745-6215-15-500 (PMC4307375; doi:10.1186/1745-6215-15-500)
Supplement: Supplementary file 1 — Additional file 1: Table S1: The simulation results of the Bayesian model for different link functions and φ Bj under the assumption of equal PPV and NPV (N = 10). Table S2. The simulation results of the Bayesian model for the different link functions and φ Bj under the assumption of higher negative predictive value (NPV) and lower positive predictive value (PPV) (N = 10). Table S3. The simulation results of the Bayesian model for the various N and φ Bj given logit link function and equal PPV and NPV. Table S4. The simulation results of the Bayesian model for the various N and φ Bj given logit link function and equal higher NPV and lower PPV. Table S5. Table of notations in the article. (DOC 887 KB) [file 13063_2014_2372_MOESM1_ESM.doc]

Additional file 1

Table S1 The simulation results of Bayesian model for different link functions and under the assumption of equal PPV and NPV (*N*=10)

|  | PPV/NPV | True value | Link function | Modified bias | Modified RMSE | Average width of 95%CIs |
| --- | --- | --- | --- | --- | --- | --- |
| 0.1 | 0.99/0.99 | 0.1404 | Odds model | -0.0115 | 0.0150 | 0.5516 |
|  |  |  | Logit model | -0.0067 | 0.0118 | 0.5256 |
|  |  |  | Cloglog model | -0.0074 | 0.0120 | 0.5173 |
|  | 0.95/0.95 | 0.2500 | Odds model | -0.0745 | 0.1141 | 0.6095 |
|  |  |  | Logit model | -0.0413 | 0.0651 | 0.5847 |
|  |  |  | Cloglog model | -0.0441 | 0.0674 | 0.5726 |
|  | 0.9/0.9 | 0.3725 | Odds model | -0.1584 | 0.2930 | 0.6400 |
|  |  |  | Logit model | -0.0706 | 0.1169 | 0.6300 |
|  |  |  | Cloglog model | -0.0806 | 0.1297 | 0.6124 |
|  | 0.8/0.8 | 0.5789 | Odds model | -0.3233 | 0.7707 | 0.6473 |
|  |  |  | Logit model | -0.0831 | 0.1472 | 0.6953 |
|  |  |  | Cloglog model | -0.1198 | 0.2028 | 0.6637 |
|  | 0.7/0.7 | 0.7460 | Odds model | -0.4775 | 1.3855 | 0.6293 |
|  |  |  | Logit model | -0.0578 | 0.1147 | 0.7467 |
|  |  |  | Cloglog model | -0.1212 | 0.2122 | 0.7022 |
|  | 0.6/0.6 | 0.8841 | Odds model | -0.6173 | 2.1247 | 0.6128 |
|  |  |  | Logit model | -0.0278 | 0.0683 | 0.7910 |
|  |  |  | Cloglog model | -0.1017 | 0.1908 | 0.7369 |
|  | 0.5/0.5 | 1.0000 | Odds model | -0.7421 | 2.9820 | 0.5960 |
|  |  |  | Logit model | -0.0418 | 0.0521 | 0.8580 |
|  |  |  | Cloglog model | -0.0794 | 0.1636 | 0.7685 |
|  | 0.25/0.25 | 1.2222 | Odds model | -1.0074 | 5.9590 | 0.5320 |
|  |  |  | Logit model | -1.2222 | . | . |
|  |  |  | Cloglog model | -0.2266 | 4.0430 | 0.0000 |
|  | 0.1/0.1 | 1.3232 | Odds model | . | . | . |
|  |  |  | Logit model | -1.3232 | . | . |
|  |  |  | Cloglog model | -0.5656 | 26.3308 | 0.0000 |
| 0.3 | 0.99/0.99 | 0.4474 | Odds model | -0.0041 | 0.0081 | 0.7680 |
|  |  |  | Logit model | 0.0122 | 0.0166 | 0.7645 |
|  |  |  | Cloglog model | 0.0200 | 0.0249 | 0.7091 |
|  | 0.95/0.95 | 0.5179 | Odds model | -0.0333 | 0.0435 | 0.7855 |
|  |  |  | Logit model | 0.0318 | 0.0438 | 0.7955 |
|  |  |  | Cloglog model | 0.0647 | 0.0767 | 0.7470 |
|  | 0.9/0.9 | 0.5966 | Odds model | -0.0741 | 0.1001 | 0.7980 |
|  |  |  | Logit model | 0.0408 | 0.0587 | 0.8183 |
|  |  |  | Cloglog model | 0.0946 | 0.1119 | 0.7751 |
|  | 0.8/0.8 | 0.7293 | Odds model | -0.1561 | 0.2338 | 0.8103 |
|  |  |  | Logit model | 0.0455 | 0.0666 | 0.8504 |
|  |  |  | Cloglog model | 0.1218 | 0.1453 | 0.8124 |
|  | 0.7/0.7 | 0.8367 | Odds model | -0.2320 | 0.3802 | 0.8139 |
|  |  |  | Logit model | 0.0408 | 0.0569 | 0.8736 |
|  |  |  | Cloglog model | 0.1309 | 0.1539 | 0.8392 |
|  | 0.6/0.6 | 0.9255 | Odds model | -0.3045 | 0.5370 | 0.8121 |
|  |  |  | Logit model | 0.0238 | 0.0353 | 0.8905 |
|  |  |  | Cloglog model | 0.1266 | 0.1465 | 0.8602 |
|  | 0.5/0.5 | 1.0000 | Odds model | -0.3760 | 0.7091 | 0.8069 |
|  |  |  | Logit model | -0.0122 | 0.0200 | 0.9072 |
|  |  |  | Cloglog model | 0.1094 | 0.1265 | 0.8763 |
|  | 0.25/0.25 | 1.1429 | Odds model | -0.5494 | 1.2501 | 0.7902 |
|  |  |  | Logit model | -1.1273 | 1175.8620 | 0.0000 |
|  |  |  | Cloglog model | -0.0487 | 1.3357 | 0.8027 |
|  | 0.1/0.1 | 1.2078 | Odds model | -0.6570 | 1.7540 | 0.7661 |
|  |  |  | Logit model | -1.2078 | 822353.0000 | . |
|  |  |  | Cloglog model | -0.2713 | 4.9100 | 0.0000 |
| 0.5 | 0.99/0.99 | 1.0000 | Odds model | 0.0088 | 0.0148 | 0.7676 |
|  |  |  | Logit model | 0.0029 | 0.0048 | 0.8941 |
|  |  |  | Cloglog model | 0.0315 | 0.0344 | 0.8352 |
|  | 0.95/0.95 | 1.0000 | Odds model | 0.0392 | 0.0548 | 0.7739 |
|  |  |  | Logit model | 0.0130 | 0.0171 | 0.8967 |
|  |  |  | Cloglog model | 0.1168 | 0.1212 | 0.8517 |
|  | 0.9/0.9 | 1.0000 | Odds model | 0.0740 | 0.1085 | 0.7782 |
|  |  |  | Logit model | 0.0215 | 0.0274 | 0.8993 |
|  |  |  | Cloglog model | 0.1785 | 0.1832 | 0.8635 |
|  | 0.8/0.8 | 1.0000 | Odds model | 0.1360 | 0.2192 | 0.7827 |
|  |  |  | Logit model | 0.0273 | 0.0350 | 0.9038 |
|  |  |  | Cloglog model | 0.2364 | 0.2406 | 0.8779 |
|  | 0.7/0.7 | 1.0000 | Odds model | 0.1953 | 0.3271 | 0.7904 |
|  |  |  | Logit model | 0.0233 | 0.0308 | 0.9068 |
|  |  |  | Cloglog model | 0.2536 | 0.2570 | 0.8864 |
|  | 0.6/0.6 | 1.0000 | Odds model | 0.2396 | 0.4132 | 0.7995 |
|  |  |  | Logit model | 0.0136 | 0.0198 | 0.9092 |
|  |  |  | Cloglog model | 0.2494 | 0.2527 | 0.8919 |
|  | 0.5/0.5 | 1.0000 | Odds model | 0.2596 | 0.4715 | 0.8074 |
|  |  |  | Logit model | 0.0072 | 0.0134 | 0.9136 |
|  |  |  | Cloglog model | 0.2344 | 0.2385 | 0.8958 |
|  | 0.25/0.25 | 1.0000 | Odds model | 0.2539 | 0.5599 | 0.8177 |
|  |  |  | Logit model | 0.0182 | 0.0352 | 0.9052 |
|  |  |  | Cloglog model | 0.1605 | 0.4390 | 0.8678 |
|  | 0.1/0.1 | 1.0000 | Odds model | 0.2154 | 0.6097 | 0.8154 |
|  |  |  | Logit model | 0.0191 | 0.0406 | 0.8916 |
|  |  |  | Cloglog model | 0.0757 | 1.0060 | 0.7897 |

Table S2 The simulation results of Bayesian model for different link functions and under the assumption of higher NPV and lower PPV (*N*=10)

|  | PPV/NPV | True value | Link function | Modified bias | Modified RMSE | Average width of 95%CIs |
| --- | --- | --- | --- | --- | --- | --- |
| 0.1 | 0.01/0.99 | 1.0000 | Odds model | -0.7689 | 3.6115 | 0.3602 |
|  |  |  | Logit model | -0.2759 | 0.4613 | 0.3878 |
|  |  |  | Cloglog model | -0.1554 | 0.4880 | 0.2536 |
|  | 0.05/0.99 | 0.5152 | Odds model | -0.3388 | 1.0277 | 0.4553 |
|  |  |  | Logit model | -0.0673 | 0.2268 | 0.2989 |
|  |  |  | Cloglog model | -0.0760 | 0.2765 | 0.2675 |
|  | 0.1/0.99 | 0.3514 | Odds model | -0.1899 | 0.4279 | 0.4861 |
|  |  |  | Logit model | -0.0519 | 0.1489 | 0.3284 |
|  |  |  | Cloglog model | -0.0563 | 0.1596 | 0.3137 |
|  | 0.25/0.99 | 0.2195 | Odds model | -0.0726 | 0.1150 | 0.5120 |
|  |  |  | Logit model | -0.0312 | 0.0702 | 0.4067 |
|  |  |  | Cloglog model | -0.0316 | 0.0705 | 0.3957 |
|  | 0.5/0.99 | 0.1677 | Odds model | -0.0309 | 0.0426 | 0.5272 |
|  |  |  | Logit model | -0.0177 | 0.0342 | 0.4700 |
|  |  |  | Cloglog model | -0.0179 | 0.0343 | 0.4612 |
|  | 0.01/0.95 | 1.2807 | Odds model | -1.0201 | 5.2266 | 0.5839 |
|  |  |  | Logit model | -0.3529 | 0.4924 | 0.7413 |
|  |  |  | Cloglog model | -0.1387 | 0.3499 | 0.5204 |
|  | 0.05/0.95 | 1.0000 | Odds model | -0.7599 | 3.2696 | 0.5982 |
|  |  |  | Logit model | -0.1346 | 0.1848 | 0.6566 |
|  |  |  | Cloglog model | -0.1004 | 0.3254 | 0.4913 |
|  | 0.1/0.95 | 0.7949 | Odds model | -0.5677 | 2.0445 | 0.6054 |
|  |  |  | Logit model | -0.0595 | 0.1506 | 0.5686 |
|  |  |  | Cloglog model | -0.0985 | 0.2709 | 0.4962 |
|  | 0.25/0.95 | 0.5152 | Odds model | -0.3060 | 0.7785 | 0.6107 |
|  |  |  | Logit model | -0.0717 | 0.1553 | 0.5450 |
|  |  |  | Cloglog model | -0.0920 | 0.1895 | 0.5204 |
|  | 0.5/0.95 | 0.3514 | Odds model | -0.1583 | 0.3018 | 0.6097 |
|  |  |  | Logit model | -0.0645 | 0.1150 | 0.5625 |
|  |  |  | Cloglog model | -0.0710 | 0.1230 | 0.5465 |
|  | 0.01/0.9 | 1.3288 | Odds model | -1.0518 | 5.2070 | 0.6346 |
|  |  |  | Logit model | -1.3288 | . | . |
|  |  |  | Cloglog model | -0.1202 | 0.2702 | 0.6277 |
|  | 0.05/0.9 | 1.1569 | Odds model | -0.8902 | 3.9757 | 0.6361 |
|  |  |  | Logit model | -0.2084 | 0.2585 | 0.7956 |
|  |  |  | Cloglog model | -0.0942 | 0.2706 | 0.5991 |
|  | 0.1/0.9 | 1.0000 | Odds model | -0.7418 | 2.9549 | 0.6362 |
|  |  |  | Logit model | -0.0926 | 0.1190 | 0.7392 |
|  |  |  | Cloglog model | -0.0928 | 0.2553 | 0.5890 |
|  | 0.25/0.9 | 0.7241 | Odds model | -0.4798 | 1.4681 | 0.6375 |
|  |  |  | Logit model | -0.0555 | 0.1362 | 0.6345 |
|  |  |  | Cloglog model | -0.1069 | 0.2259 | 0.5930 |
|  | 0.5/0.9 | 0.5152 | Odds model | -0.2846 | 0.6636 | 0.6394 |
|  |  |  | Logit model | -0.0796 | 0.1480 | 0.6273 |
|  |  |  | Cloglog model | -0.1032 | 0.1833 | 0.6010 |
| 0.3 | 0.01/0.99 | 1.0000 | Odds model | -0.2140 | 0.7301 | 0.3439 |
|  |  |  | Logit model | -0.1229 | 0.1695 | 0.6773 |
|  |  |  | Cloglog model | 0.1911 | 0.2971 | 0.4968 |
|  | 0.05/0.99 | 0.6883 | Odds model | -0.1184 | 0.2490 | 0.4954 |
|  |  |  | Logit model | 0.0384 | 0.1077 | 0.6206 |
|  |  |  | Cloglog model | 0.1690 | 0.2447 | 0.5064 |
|  | 0.1/0.99 | 0.5830 | Odds model | -0.0688 | 0.1305 | 0.5791 |
|  |  |  | Logit model | 0.0409 | 0.0884 | 0.6430 |
|  |  |  | Cloglog model | 0.1181 | 0.1687 | 0.5469 |
|  | 0.25/0.99 | 0.4983 | Odds model | -0.0260 | 0.0533 | 0.6763 |
|  |  |  | Logit model | 0.0254 | 0.0540 | 0.6959 |
|  |  |  | Cloglog model | 0.0590 | 0.0893 | 0.6163 |
|  | 0.5/0.99 | 0.4650 | Odds model | -0.0104 | 0.0268 | 0.7303 |
|  |  |  | Logit model | 0.0154 | 0.0319 | 0.7337 |
|  |  |  | Cloglog model | 0.0316 | 0.0496 | 0.6678 |
|  | 0.01/0.95 | 1.1805 | Odds model | -0.4324 | 1.0754 | 0.5775 |
|  |  |  | Logit model | -0.2068 | 0.2525 | 0.8511 |
|  |  |  | Cloglog model | 0.1906 | 0.2399 | 0.7130 |
|  | 0.05/0.95 | 1.0000 | Odds model | -0.3327 | 0.7016 | 0.6228 |
|  |  |  | Logit model | -0.0531 | 0.0684 | 0.8212 |
|  |  |  | Cloglog model | 0.2189 | 0.2690 | 0.6979 |
|  | 0.1/0.95 | 0.8681 | Odds model | -0.2542 | 0.4699 | 0.6604 |
|  |  |  | Logit model | 0.0211 | 0.0594 | 0.7869 |
|  |  |  | Cloglog model | 0.2068 | 0.2486 | 0.6997 |
|  | 0.25/0.95 | 0.6883 | Odds model | -0.1402 | 0.2132 | 0.7210 |
|  |  |  | Logit model | 0.0461 | 0.0768 | 0.7773 |
|  |  |  | Cloglog model | 0.1502 | 0.1815 | 0.7129 |
|  | 0.5/0.95 | 0.5830 | Odds model | -0.0717 | 0.0984 | 0.7591 |
|  |  |  | Logit model | 0.0389 | 0.0611 | 0.7850 |
|  |  |  | Cloglog model | 0.0982 | 0.1195 | 0.7297 |
|  | 0.01/0.9 | 1.2114 | Odds model | -0.4894 | 1.1700 | 0.6785 |
|  |  |  | Logit model | -0.3644 | 7.2938 | 0.0000 |
|  |  |  | Cloglog model | 0.1672 | 0.2000 | 0.7798 |
|  | 0.05/0.9 | 1.1008 | Odds model | -0.4143 | 0.9020 | 0.6967 |
|  |  |  | Logit model | -0.1177 | 0.1339 | 0.8719 |
|  |  |  | Cloglog model | 0.1940 | 0.2283 | 0.7692 |
|  | 0.1/0.9 | 1.0000 | Odds model | -0.3467 | 0.6910 | 0.7141 |
|  |  |  | Logit model | -0.0340 | 0.0440 | 0.8560 |
|  |  |  | Cloglog model | 0.2006 | 0.2328 | 0.7651 |
|  | 0.25/0.9 | 0.8227 | Odds model | -0.2270 | 0.3816 | 0.7501 |
|  |  |  | Logit model | 0.0390 | 0.0641 | 0.8221 |
|  |  |  | Cloglog model | 0.1753 | 0.2051 | 0.7660 |
|  | 0.5/0.9 | 0.6883 | Odds model | -0.1345 | 0.1975 | 0.7786 |
|  |  |  | Logit model | 0.0454 | 0.0695 | 0.8182 |
|  |  |  | Cloglog model | 0.1311 | 0.1556 | 0.7693 |
| 0.5 | 0.01/0.99 | 1.0000 | Odds model | 0.7817 | 1.1601 | 0.3248 |
|  |  |  | Logit model | 0.0023 | 0.0046 | 0.8844 |
|  |  |  | Cloglog model | 0.4847 | 0.4961 | 0.7305 |
|  | 0.05/0.99 | 1.0000 | Odds model | 0.2764 | 0.4225 | 0.4735 |
|  |  |  | Logit model | 0.0035 | 0.0068 | 0.8852 |
|  |  |  | Cloglog model | 0.3083 | 0.3255 | 0.7390 |
|  | 0.1/0.99 | 1.0000 | Odds model | 0.1473 | 0.2382 | 0.5597 |
|  |  |  | Logit model | 0.0047 | 0.0086 | 0.8863 |
|  |  |  | Cloglog model | 0.2104 | 0.2260 | 0.7587 |
|  | 0.25/0.99 | 1.0000 | Odds model | 0.0566 | 0.1100 | 0.6651 |
|  |  |  | Logit model | 0.0052 | 0.0100 | 0.8886 |
|  |  |  | Cloglog model | 0.1064 | 0.1196 | 0.7921 |
|  | 0.5/0.99 | 1.0000 | Odds model | 0.0258 | 0.0586 | 0.7259 |
|  |  |  | Logit model | 0.0044 | 0.0089 | 0.8912 |
|  |  |  | Cloglog model | 0.0572 | 0.0667 | 0.8161 |
|  | 0.01/0.95 | 1.0000 | Odds model | 0.6080 | 1.0229 | 0.5331 |
|  |  |  | Logit model | 0.0046 | 0.0107 | 0.8872 |
|  |  |  | Cloglog model | 0.5292 | 0.5329 | 0.8215 |
|  | 0.05/0.95 | 1.0000 | Odds model | 0.4263 | 0.6889 | 0.5810 |
|  |  |  | Logit model | 0.0045 | 0.0092 | 0.8880 |
|  |  |  | Cloglog model | 0.4623 | 0.4676 | 0.8222 |
|  | 0.1/0.95 | 1.0000 | Odds model | 0.3031 | 0.4784 | 0.6210 |
|  |  |  | Logit model | 0.0064 | 0.0113 | 0.8887 |
|  |  |  | Cloglog model | 0.3967 | 0.4024 | 0.8258 |
|  | 0.25/0.95 | 1.0000 | Odds model | 0.1539 | 0.2353 | 0.6897 |
|  |  |  | Logit model | 0.0129 | 0.0189 | 0.8913 |
|  |  |  | Cloglog model | 0.2763 | 0.2828 | 0.8344 |
|  | 0.5/0.95 | 1.0000 | Odds model | 0.0811 | 0.1207 | 0.7386 |
|  |  |  | Logit model | 0.0151 | 0.0207 | 0.8941 |
|  |  |  | Cloglog model | 0.1833 | 0.1895 | 0.8432 |
|  | 0.01/0.9 | 1.0000 | Odds model | 0.4881 | 0.9475 | 0.6266 |
|  |  |  | Logit model | 0.0072 | 0.0179 | 0.8905 |
|  |  |  | Cloglog model | 0.4888 | 0.4920 | 0.8442 |
|  | 0.05/0.9 | 1.0000 | Odds model | 0.4202 | 0.7554 | 0.6479 |
|  |  |  | Logit model | 0.0061 | 0.0143 | 0.8913 |
|  |  |  | Cloglog model | 0.4539 | 0.4575 | 0.8458 |
|  | 0.1/0.9 | 1.0000 | Odds model | 0.3490 | 0.6008 | 0.6683 |
|  |  |  | Logit model | 0.0060 | 0.0121 | 0.8921 |
|  |  |  | Cloglog model | 0.4162 | 0.4199 | 0.8480 |
|  | 0.25/0.9 | 1.0000 | Odds model | 0.2190 | 0.3561 | 0.7114 |
|  |  |  | Logit model | 0.0131 | 0.0199 | 0.8939 |
|  |  |  | Cloglog model | 0.3312 | 0.3357 | 0.8537 |
|  | 0.5/0.9 | 1.0000 | Odds model | 0.1299 | 0.2009 | 0.7493 |
|  |  |  | Logit model | 0.0208 | 0.0278 | 0.8970 |
|  |  |  | Cloglog model | 0.2475 | 0.2527 | 0.8591 |

Table S3 The simulation results of Bayesian model for various *N* and given logit link function and equal PPV and NPV

|  | PPV/NPV | True value | *N* | Modified bias | Modified RMSE | Average width of 95%CIs |
| --- | --- | --- | --- | --- | --- | --- |
| 0.1 | 0.99/0.99 | 0.1404 | 5 | -0.0066 | 0.0151 | 0.3867 |
|  |  | 0.1404 | 10 | -0.0067 | 0.0118 | 0.5256 |
|  |  | 0.1404 | 20 | -0.0064 | 0.0094 | 0.6361 |
|  |  | 0.1404 | 30 | -0.0062 | 0.0083 | 0.6917 |
|  |  | 0.1404 | 50 | -0.0061 | 0.0075 | 0.7507 |
|  | 0.95/0.95 | 0.2500 | 5 | -0.0449 | 0.0816 | 0.4509 |
|  |  | 0.2500 | 10 | -0.0413 | 0.0651 | 0.5847 |
|  |  | 0.2500 | 20 | -0.0389 | 0.0542 | 0.6859 |
|  |  | 0.2500 | 30 | -0.0377 | 0.0500 | 0.7361 |
|  |  | 0.2500 | 50 | -0.0367 | 0.0466 | 0.7881 |
|  | 0.9/0.9 | 0.3725 | 5 | -0.0797 | 0.1524 | 0.5023 |
|  |  | 0.3725 | 10 | -0.0706 | 0.1169 | 0.6300 |
|  |  | 0.3725 | 20 | -0.0656 | 0.0951 | 0.7234 |
|  |  | 0.3725 | 30 | -0.0630 | 0.0864 | 0.7688 |
|  |  | 0.3725 | 50 | -0.0613 | 0.0800 | 0.8159 |
|  | 0.8/0.8 | 0.5789 | 5 | -0.1039 | 0.2080 | 0.5771 |
|  |  | 0.5789 | 10 | -0.0831 | 0.1472 | 0.6953 |
|  |  | 0.5789 | 20 | -0.0732 | 0.1108 | 0.7764 |
|  |  | 0.5789 | 30 | -0.0690 | 0.0970 | 0.8150 |
|  |  | 0.5789 | 50 | -0.0665 | 0.0871 | 0.8538 |
|  | 0.7/0.7 | 0.7460 | 5 | -0.0851 | 0.1774 | 0.6371 |
|  |  | 0.7460 | 10 | -0.0578 | 0.1147 | 0.7467 |
|  |  | 0.7460 | 20 | -0.0461 | 0.0800 | 0.8176 |
|  |  | 0.7460 | 30 | -0.0418 | 0.0672 | 0.8501 |
|  |  | 0.7460 | 50 | -0.0393 | 0.0564 | 0.8821 |
|  | 0.6/0.6 | 0.8841 | 5 | -0.0577 | 0.1164 | 0.7002 |
|  |  | 0.8841 | 10 | -0.0278 | 0.0683 | 0.7910 |
|  |  | 0.8841 | 20 | -0.0163 | 0.0434 | 0.8499 |
|  |  | 0.8841 | 30 | -0.0133 | 0.0355 | 0.8773 |
|  |  | 0.8841 | 50 | -0.0114 | 0.0272 | 0.9041 |
|  | 0.5/0.5 | 1.0000 | 5 | -0.0688 | 0.0891 | 0.7902 |
|  |  | 1.0000 | 10 | -0.0418 | 0.0521 | 0.8580 |
|  |  | 1.0000 | 20 | -0.0264 | 0.0325 | 0.8993 |
|  |  | 1.0000 | 30 | -0.0207 | 0.0257 | 0.9172 |
|  |  | 1.0000 | 50 | -0.0154 | 0.0194 | 0.9350 |
| 0.3 | 0.99/0.99 | 0.4474 | 5 | 0.0102 | 0.0188 | 0.6821 |
|  |  | 0.4474 | 10 | 0.0122 | 0.0166 | 0.7645 |
|  |  | 0.4474 | 20 | 0.0138 | 0.0160 | 0.8206 |
|  |  | 0.4474 | 30 | 0.0147 | 0.0161 | 0.8468 |
|  |  | 0.4474 | 50 | 0.0156 | 0.0165 | 0.8744 |
|  | 0.95/0.95 | 0.5179 | 5 | 0.0243 | 0.0491 | 0.7225 |
|  |  | 0.5179 | 10 | 0.0318 | 0.0438 | 0.7955 |
|  |  | 0.5179 | 20 | 0.0380 | 0.0434 | 0.8443 |
|  |  | 0.5179 | 30 | 0.0417 | 0.0451 | 0.8678 |
|  |  | 0.5179 | 50 | 0.0456 | 0.0478 | 0.8921 |
|  | 0.9/0.9 | 0.5966 | 5 | 0.0276 | 0.0660 | 0.7525 |
|  |  | 0.5966 | 10 | 0.0408 | 0.0587 | 0.8183 |
|  |  | 0.5966 | 20 | 0.0511 | 0.0588 | 0.8624 |
|  |  | 0.5966 | 30 | 0.0570 | 0.0618 | 0.8836 |
|  |  | 0.5966 | 50 | 0.0631 | 0.0659 | 0.9057 |
|  | 0.8/0.8 | 0.7293 | 5 | 0.0253 | 0.0743 | 0.7929 |
|  |  | 0.7293 | 10 | 0.0455 | 0.0666 | 0.8504 |
|  |  | 0.7293 | 20 | 0.0597 | 0.0683 | 0.8879 |
|  |  | 0.7293 | 30 | 0.0670 | 0.0722 | 0.9058 |
|  |  | 0.7293 | 50 | 0.0743 | 0.0773 | 0.9243 |
|  | 0.7/0.7 | 0.8367 | 5 | 0.0204 | 0.0630 | 0.8228 |
|  |  | 0.8367 | 10 | 0.0408 | 0.0569 | 0.8736 |
|  |  | 0.8367 | 20 | 0.0534 | 0.0598 | 0.9060 |
|  |  | 0.8367 | 30 | 0.0593 | 0.0633 | 0.9212 |
|  |  | 0.8367 | 50 | 0.0649 | 0.0672 | 0.9368 |
|  | 0.6/0.6 | 0.9255 | 5 | 0.0072 | 0.0404 | 0.8486 |
|  |  | 0.9255 | 10 | 0.0238 | 0.0353 | 0.8905 |
|  |  | 0.9255 | 20 | 0.0322 | 0.0366 | 0.9181 |
|  |  | 0.9255 | 30 | 0.0354 | 0.0383 | 0.9311 |
|  |  | 0.9255 | 50 | 0.0383 | 0.0400 | 0.9447 |
|  | 0.5/0.5 | 1.0000 | 5 | -0.0239 | 0.0337 | 0.8781 |
|  |  | 1.0000 | 10 | -0.0122 | 0.0200 | 0.9072 |
|  |  | 1.0000 | 20 | -0.0062 | 0.0134 | 0.9279 |
|  |  | 1.0000 | 30 | -0.0043 | 0.0113 | 0.9383 |
|  |  | 1.0000 | 50 | -0.0028 | 0.0094 | 0.9499 |
| 0.5 | 0.99/0.99 | 1.0000 | 5 | 0.0015 | 0.0031 | 0.8886 |
|  |  | 1.0000 | 10 | 0.0029 | 0.0048 | 0.8941 |
|  |  | 1.0000 | 20 | 0.0046 | 0.0063 | 0.9033 |
|  |  | 1.0000 | 30 | 0.0061 | 0.0075 | 0.9103 |
|  |  | 1.0000 | 50 | 0.0080 | 0.0091 | 0.9207 |
|  | 0.95/0.95 | 1.0000 | 5 | 0.0060 | 0.0101 | 0.8899 |
|  |  | 1.0000 | 10 | 0.0130 | 0.0171 | 0.8967 |
|  |  | 1.0000 | 20 | 0.0226 | 0.0256 | 0.9076 |
|  |  | 1.0000 | 30 | 0.0291 | 0.0315 | 0.9153 |
|  |  | 1.0000 | 50 | 0.0375 | 0.0393 | 0.9263 |
|  | 0.9/0.9 | 1.0000 | 5 | 0.0101 | 0.0163 | 0.8913 |
|  |  | 1.0000 | 10 | 0.0215 | 0.0274 | 0.8993 |
|  |  | 1.0000 | 20 | 0.0363 | 0.0404 | 0.9114 |
|  |  | 1.0000 | 30 | 0.0458 | 0.0490 | 0.9199 |
|  |  | 1.0000 | 50 | 0.0577 | 0.0599 | 0.9313 |
|  | 0.8/0.8 | 1.0000 | 5 | 0.0132 | 0.0217 | 0.8939 |
|  |  | 1.0000 | 10 | 0.0273 | 0.0350 | 0.9038 |
|  |  | 1.0000 | 20 | 0.0441 | 0.0493 | 0.9173 |
|  |  | 1.0000 | 30 | 0.0541 | 0.0581 | 0.9262 |
|  |  | 1.0000 | 50 | 0.0662 | 0.0689 | 0.9378 |
|  | 0.7/0.7 | 1.0000 | 5 | 0.0120 | 0.0203 | 0.8960 |
|  |  | 1.0000 | 10 | 0.0233 | 0.0308 | 0.9068 |
|  |  | 1.0000 | 20 | 0.0356 | 0.0409 | 0.9209 |
|  |  | 1.0000 | 30 | 0.0427 | 0.0468 | 0.9300 |
|  |  | 1.0000 | 50 | 0.0509 | 0.0536 | 0.9415 |
|  | 0.6/0.6 | 1.0000 | 5 | 0.0083 | 0.0146 | 0.8983 |
|  |  | 1.0000 | 10 | 0.0136 | 0.0198 | 0.9093 |
|  |  | 1.0000 | 20 | 0.0192 | 0.0239 | 0.9233 |
|  |  | 1.0000 | 30 | 0.0223 | 0.0262 | 0.9324 |
|  |  | 1.0000 | 50 | 0.0259 | 0.0286 | 0.9438 |
|  | 0.5/0.5 | 1.0000 | 5 | 0.0062 | 0.0126 | 0.9015 |
|  |  | 1.0000 | 10 | 0.0072 | 0.0134 | 0.9136 |
|  |  | 1.0000 | 20 | 0.0069 | 0.0125 | 0.9284 |
|  |  | 1.0000 | 30 | 0.0063 | 0.0115 | 0.9376 |
|  |  | 1.0000 | 50 | 0.0052 | 0.0100 | 0.9486 |

Table S4 The simulation results of Bayesian model for various *N* and given logit link function and equal higher NPV and lower PPV

|  | PPV/NPV | True value | *N* | Modified bias | Modified RMSE | Average width of 95%CIs |
| --- | --- | --- | --- | --- | --- | --- |
| 0.1 | 0.01/0.99 | 1.0000 | 5 | -0.4017 | 0.8863 | 0.1890 |
|  |  |  | 10 | -0.2759 | 0.4613 | 0.3878 |
|  |  |  | 20 | -0.1931 | 0.2753 | 0.5410 |
|  |  |  | 30 | -0.1555 | 0.2095 | 0.6150 |
|  |  |  | 50 | -0.1203 | 0.1562 | 0.6881 |
|  | 0.05/0.99 | 0.5152 | 5 | -0.1122 | 0.3575 | 0.1647 |
|  |  |  | 10 | -0.0673 | 0.2268 | 0.2989 |
|  |  |  | 20 | -0.0521 | 0.1593 | 0.4270 |
|  |  |  | 30 | -0.0452 | 0.1273 | 0.5027 |
|  |  |  | 50 | -0.0416 | 0.1009 | 0.5884 |
|  | 0.1/0.99 | 0.3514 | 5 | -0.0634 | 0.2192 | 0.1876 |
|  |  |  | 10 | -0.0519 | 0.1489 | 0.3284 |
|  |  |  | 20 | -0.0480 | 0.1101 | 0.4620 |
|  |  |  | 30 | -0.0457 | 0.0923 | 0.5349 |
|  |  |  | 50 | -0.0429 | 0.0778 | 0.6163 |
|  | 0.25/0.99 | 0.2195 | 5 | -0.0328 | 0.0962 | 0.2608 |
|  |  |  | 10 | -0.0312 | 0.0702 | 0.4067 |
|  |  |  | 20 | -0.0300 | 0.0538 | 0.5319 |
|  |  |  | 30 | -0.0296 | 0.0474 | 0.5981 |
|  |  |  | 50 | -0.0285 | 0.0418 | 0.6714 |
|  | 0.5/0.99 | 0.1677 | 5 | -0.0177 | 0.0455 | 0.3272 |
|  |  |  | 10 | -0.0177 | 0.0343 | 0.4700 |
|  |  |  | 20 | -0.0167 | 0.0266 | 0.5886 |
|  |  |  | 30 | -0.0162 | 0.0237 | 0.6491 |
|  |  |  | 50 | -0.0157 | 0.0212 | 0.7150 |
|  | 0.01/0.95 | 1.2807 | 5 | -0.4271 | 0.6825 | 0.5736 |
|  |  |  | 10 | -0.3529 | 0.4924 | 0.7413 |
|  |  |  | 20 | -0.3151 | 0.4196 | 0.8280 |
|  |  |  | 30 | -0.3005 | 0.3939 | 0.8590 |
|  |  |  | 50 | -0.2872 | 0.3714 | 0.8844 |
|  | 0.05/0.95 | 1.0000 | 5 | -0.2151 | 0.3449 | 0.4962 |
|  |  |  | 10 | -0.1346 | 0.1848 | 0.6566 |
|  |  |  | 20 | -0.0886 | 0.1117 | 0.7517 |
|  |  |  | 30 | -0.0707 | 0.0887 | 0.7916 |
|  |  |  | 50 | -0.0534 | 0.0667 | 0.8309 |
|  | 0.1/0.95 | 0.7949 | 5 | -0.1223 | 0.2703 | 0.4267 |
|  |  |  | 10 | -0.0595 | 0.1505 | 0.5686 |
|  |  |  | 20 | -0.0319 | 0.0986 | 0.6655 |
|  |  |  | 30 | -0.0249 | 0.0827 | 0.7152 |
|  |  |  | 50 | -0.0197 | 0.0656 | 0.7697 |
|  | 0.25/0.95 | 0.5152 | 5 | -0.0940 | 0.2307 | 0.3969 |
|  |  |  | 10 | -0.0717 | 0.1553 | 0.5451 |
|  |  |  | 20 | -0.0596 | 0.1099 | 0.6564 |
|  |  |  | 30 | -0.0559 | 0.0939 | 0.7108 |
|  |  |  | 50 | -0.0522 | 0.0799 | 0.7680 |
|  | 0.5/0.95 | 0.3514 | 5 | -0.0733 | 0.1527 | 0.4232 |
|  |  |  | 10 | -0.0645 | 0.1150 | 0.5625 |
|  |  |  | 20 | -0.0581 | 0.0893 | 0.6683 |
|  |  |  | 30 | -0.0557 | 0.0800 | 0.7206 |
|  |  |  | 50 | -0.0536 | 0.0726 | 0.7759 |
|  | 0.01/0.9 | 1.3288 | 5 | -1.3288 | . | . |
|  |  |  | 10 | -1.3288 | . | . |
|  |  |  | 20 | -0.3385 | 0.4558 | 0.8759 |
|  |  |  | 30 | -0.3732 | 4.4908 | . |
|  |  |  | 50 | -0.3196 | 0.4226 | 0.9066 |
|  | 0.05/0.9 | 1.1569 | 5 | -0.2640 | 0.3635 | 0.6683 |
|  |  |  | 10 | -0.2084 | 0.2584 | 0.7956 |
|  |  |  | 20 | -0.1804 | 0.2158 | 0.8581 |
|  |  |  | 30 | -0.1693 | 0.2000 | 0.8817 |
|  |  |  | 50 | -0.1597 | 0.1869 | 0.9029 |
|  | 0.1/0.9 | 1.0000 | 5 | -0.1549 | 0.2283 | 0.6093 |
|  |  |  | 10 | -0.0926 | 0.1190 | 0.7392 |
|  |  |  | 20 | -0.0605 | 0.0754 | 0.8106 |
|  |  |  | 30 | -0.0471 | 0.0586 | 0.8414 |
|  |  |  | 50 | -0.0353 | 0.0450 | 0.8718 |
|  | 0.25/0.9 | 0.7241 | 5 | -0.0931 | 0.2227 | 0.5019 |
|  |  |  | 10 | -0.0555 | 0.1362 | 0.6345 |
|  |  |  | 20 | -0.0397 | 0.0906 | 0.7303 |
|  |  |  | 30 | -0.0349 | 0.0734 | 0.7762 |
|  |  |  | 50 | -0.0313 | 0.0599 | 0.8224 |
|  | 0.5/0.9 | 0.5152 | 5 | -0.0986 | 0.2092 | 0.4941 |
|  |  |  | 10 | -0.0796 | 0.1481 | 0.6273 |
|  |  |  | 20 | -0.0687 | 0.1093 | 0.7226 |
|  |  |  | 30 | -0.0645 | 0.0947 | 0.7688 |
|  |  |  | 50 | -0.0616 | 0.0845 | 0.8158 |
| 0.3 | 0.01/0.99 | 1.0000 | 5 | -0.1987 | 0.3311 | 0.5163 |
|  |  |  | 10 | -0.1229 | 0.1695 | 0.6773 |
|  |  |  | 20 | -0.0806 | 0.1014 | 0.7663 |
|  |  |  | 30 | -0.0629 | 0.0774 | 0.8026 |
|  |  |  | 50 | -0.0467 | 0.0576 | 0.8351 |
|  | 0.05/0.99 | 0.6883 | 5 | -0.0016 | 0.1518 | 0.4938 |
|  |  |  | 10 | 0.0384 | 0.1077 | 0.6206 |
|  |  |  | 20 | 0.0536 | 0.0897 | 0.7106 |
|  |  |  | 30 | 0.0606 | 0.0839 | 0.7551 |
|  |  |  | 50 | 0.0663 | 0.0804 | 0.8002 |
|  | 0.1/0.99 | 0.5830 | 5 | 0.0230 | 0.1184 | 0.5196 |
|  |  |  | 10 | 0.0409 | 0.0884 | 0.6430 |
|  |  |  | 20 | 0.0490 | 0.0746 | 0.7309 |
|  |  |  | 30 | 0.0534 | 0.0703 | 0.7717 |
|  |  |  | 50 | 0.0591 | 0.0699 | 0.8129 |
|  | 0.25/0.99 | 0.4983 | 5 | 0.0188 | 0.0715 | 0.5886 |
|  |  |  | 10 | 0.0254 | 0.0540 | 0.6959 |
|  |  |  | 20 | 0.0298 | 0.0446 | 0.7687 |
|  |  |  | 30 | 0.0320 | 0.0419 | 0.8027 |
|  |  |  | 50 | 0.0355 | 0.0418 | 0.8379 |
|  | 0.5/0.99 | 0.4650 | 5 | 0.0122 | 0.0422 | 0.6408 |
|  |  |  | 10 | 0.0154 | 0.0319 | 0.7337 |
|  |  |  | 20 | 0.0188 | 0.0272 | 0.7974 |
|  |  |  | 30 | 0.0204 | 0.0260 | 0.8269 |
|  |  |  | 50 | 0.0223 | 0.0260 | 0.8578 |
|  | 0.01/0.95 | 1.1805 | 5 | -0.2408 | 0.3138 | 0.7784 |
|  |  |  | 10 | -0.2068 | 0.2525 | 0.8511 |
|  |  |  | 20 | -0.1897 | 0.2271 | 0.8801 |
|  |  |  | 30 | -0.1828 | 0.2174 | 0.8895 |
|  |  |  | 50 | -0.1759 | 0.2080 | 0.8985 |
|  | 0.05/0.95 | 1.0000 | 5 | -0.0919 | 0.1286 | 0.7416 |
|  |  |  | 10 | -0.0531 | 0.0684 | 0.8212 |
|  |  |  | 20 | -0.0320 | 0.0409 | 0.8612 |
|  |  |  | 30 | -0.0240 | 0.0328 | 0.8769 |
|  |  |  | 50 | -0.0164 | 0.0255 | 0.8928 |
|  | 0.1/0.95 | 0.8681 | 5 | -0.0138 | 0.0919 | 0.7059 |
|  |  |  | 10 | 0.0211 | 0.0594 | 0.7869 |
|  |  |  | 20 | 0.0377 | 0.0543 | 0.8355 |
|  |  |  | 30 | 0.0433 | 0.0547 | 0.8584 |
|  |  |  | 50 | 0.0490 | 0.0562 | 0.8823 |
|  | 0.25/0.95 | 0.6883 | 5 | 0.0256 | 0.0942 | 0.6902 |
|  |  |  | 10 | 0.0461 | 0.0768 | 0.7773 |
|  |  |  | 20 | 0.0599 | 0.0731 | 0.8326 |
|  |  |  | 30 | 0.0663 | 0.0747 | 0.8578 |
|  |  |  | 50 | 0.0742 | 0.0794 | 0.8835 |
|  | 0.5/0.95 | 0.5830 | 5 | 0.0264 | 0.0719 | 0.7062 |
|  |  |  | 10 | 0.0389 | 0.0611 | 0.7850 |
|  |  |  | 20 | 0.0496 | 0.0593 | 0.8371 |
|  |  |  | 30 | 0.0551 | 0.0613 | 0.8614 |
|  |  |  | 50 | 0.0614 | 0.0654 | 0.8869 |
|  | 0.01/0.9 | 1.2114 | 5 | -0.3926 | 9.0453 | . |
|  |  |  | 10 | -0.3644 | 7.2938 | 0.0000 |
|  |  |  | 20 | -0.2084 | 0.2534 | 0.8942 |
|  |  |  | 30 | -0.2242 | 1.2616 | 0.0000 |
|  |  |  | 50 | -0.1968 | 0.2373 | 0.9102 |
|  | 0.05/0.9 | 1.1008 | 5 | -0.1426 | 0.1704 | 0.8223 |
|  |  |  | 10 | -0.1177 | 0.1339 | 0.8719 |
|  |  |  | 20 | -0.1048 | 0.1178 | 0.8928 |
|  |  |  | 30 | -0.0995 | 0.1114 | 0.9018 |
|  |  |  | 50 | -0.0948 | 0.1059 | 0.9124 |
|  | 0.1/0.9 | 1.0000 | 5 | -0.0626 | 0.0854 | 0.7990 |
|  |  |  | 10 | -0.0340 | 0.0440 | 0.8560 |
|  |  |  | 20 | -0.0194 | 0.0282 | 0.8846 |
|  |  |  | 30 | -0.0135 | 0.0229 | 0.8974 |
|  |  |  | 50 | -0.0087 | 0.0191 | 0.9114 |
|  | 0.25/0.9 | 0.8227 | 5 | 0.0146 | 0.0804 | 0.7527 |
|  |  |  | 10 | 0.0390 | 0.0641 | 0.8221 |
|  |  |  | 20 | 0.0525 | 0.0629 | 0.8663 |
|  |  |  | 30 | 0.0585 | 0.0649 | 0.8865 |
|  |  |  | 50 | 0.0652 | 0.0692 | 0.9070 |
|  | 0.5/0.9 | 0.6883 | 5 | 0.0265 | 0.0805 | 0.7486 |
|  |  |  | 10 | 0.0454 | 0.0695 | 0.8182 |
|  |  |  | 20 | 0.0598 | 0.0698 | 0.8631 |
|  |  |  | 30 | 0.0671 | 0.0731 | 0.8842 |
|  |  |  | 50 | 0.0748 | 0.0787 | 0.9058 |
| 0.5 | 0.01/0.99 | 1.0000 | 5 | 0.0017 | 0.0034 | 0.8838 |
|  |  |  | 10 | 0.0023 | 0.0046 | 0.8844 |
|  |  |  | 20 | 0.0031 | 0.0064 | 0.8853 |
|  |  |  | 30 | 0.0035 | 0.0076 | 0.8866 |
|  |  |  | 50 | 0.0045 | 0.0096 | 0.8882 |
|  | 0.05/0.99 | 1.0000 | 5 | 0.0018 | 0.0043 | 0.8842 |
|  |  |  | 10 | 0.0035 | 0.0068 | 0.8852 |
|  |  |  | 20 | 0.0066 | 0.0105 | 0.8872 |
|  |  |  | 30 | 0.0095 | 0.0138 | 0.8891 |
|  |  |  | 50 | 0.0147 | 0.0191 | 0.8924 |
|  | 0.1/0.99 | 1.0000 | 5 | 0.0022 | 0.0055 | 0.8847 |
|  |  |  | 10 | 0.0047 | 0.0086 | 0.8863 |
|  |  |  | 20 | 0.0093 | 0.0138 | 0.8893 |
|  |  |  | 30 | 0.0135 | 0.0183 | 0.8921 |
|  |  |  | 50 | 0.0203 | 0.0249 | 0.8969 |
|  | 0.25/0.99 | 1.0000 | 5 | 0.0026 | 0.0065 | 0.8858 |
|  |  |  | 10 | 0.0052 | 0.0100 | 0.8886 |
|  |  |  | 20 | 0.0097 | 0.0149 | 0.8938 |
|  |  |  | 30 | 0.0135 | 0.0185 | 0.8982 |
|  |  |  | 50 | 0.0192 | 0.0236 | 0.9057 |
|  | 0.5/0.99 | 1.0000 | 5 | 0.0022 | 0.0060 | 0.8872 |
|  |  |  | 10 | 0.0044 | 0.0089 | 0.8912 |
|  |  |  | 20 | 0.0077 | 0.0125 | 0.8984 |
|  |  |  | 30 | 0.0103 | 0.0147 | 0.9042 |
|  |  |  | 50 | 0.0137 | 0.0176 | 0.9134 |
|  | 0.01/0.95 | 1.0000 | 5 | 0.0032 | 0.0079 | 0.8852 |
|  |  |  | 10 | 0.0046 | 0.0107 | 0.8872 |
|  |  |  | 20 | 0.0072 | 0.0151 | 0.8909 |
|  |  |  | 30 | 0.0090 | 0.0177 | 0.8944 |
|  |  |  | 50 | 0.0117 | 0.0208 | 0.9000 |
|  | 0.05/0.95 | 1.0000 | 5 | 0.0030 | 0.0067 | 0.8856 |
|  |  |  | 10 | 0.0045 | 0.0092 | 0.8880 |
|  |  |  | 20 | 0.0063 | 0.0124 | 0.8922 |
|  |  |  | 30 | 0.0071 | 0.0141 | 0.8960 |
|  |  |  | 50 | 0.0079 | 0.0158 | 0.9024 |
|  | 0.1/0.95 | 1.0000 | 5 | 0.0037 | 0.0077 | 0.8861 |
|  |  |  | 10 | 0.0064 | 0.0113 | 0.8887 |
|  |  |  | 20 | 0.0111 | 0.0169 | 0.8935 |
|  |  |  | 30 | 0.0147 | 0.0205 | 0.8976 |
|  |  |  | 50 | 0.0202 | 0.0259 | 0.9045 |
|  | 0.25/0.95 | 1.0000 | 5 | 0.0060 | 0.0111 | 0.8871 |
|  |  |  | 10 | 0.0129 | 0.0189 | 0.8913 |
|  |  |  | 20 | 0.0239 | 0.0295 | 0.8985 |
|  |  |  | 30 | 0.0321 | 0.0371 | 0.9043 |
|  |  |  | 50 | 0.0439 | 0.0480 | 0.9134 |
|  | 0.5/0.95 | 1.0000 | 5 | 0.0069 | 0.0122 | 0.8885 |
|  |  |  | 10 | 0.0151 | 0.0207 | 0.8941 |
|  |  |  | 20 | 0.0272 | 0.0320 | 0.9033 |
|  |  |  | 30 | 0.0357 | 0.0397 | 0.9104 |
|  |  |  | 50 | 0.0470 | 0.0501 | 0.9208 |
|  | 0.01/0.9 | 1.0000 | 5 | 0.0050 | 0.0130 | 0.8869 |
|  |  |  | 10 | 0.0072 | 0.0179 | 0.8905 |
|  |  |  | 20 | 0.0111 | 0.0239 | 0.8967 |
|  |  |  | 30 | 0.0142 | 0.0270 | 0.9021 |
|  |  |  | 50 | 0.0179 | 0.0298 | 0.9103 |
|  | 0.05/0.9 | 1.0000 | 5 | 0.0043 | 0.0106 | 0.8874 |
|  |  |  | 10 | 0.0061 | 0.0143 | 0.8913 |
|  |  |  | 20 | 0.0087 | 0.0183 | 0.8982 |
|  |  |  | 30 | 0.0102 | 0.0202 | 0.9039 |
|  |  |  | 50 | 0.0116 | 0.0211 | 0.9128 |
|  | 0.1/0.9 | 1.0000 | 5 | 0.0044 | 0.0092 | 0.8878 |
|  |  |  | 10 | 0.0060 | 0.0121 | 0.8921 |
|  |  |  | 20 | 0.0079 | 0.0155 | 0.8992 |
|  |  |  | 30 | 0.0085 | 0.0166 | 0.9052 |
|  |  |  | 50 | 0.0085 | 0.0168 | 0.9142 |
|  | 0.25/0.9 | 1.0000 | 5 | 0.0068 | 0.0124 | 0.8886 |
|  |  |  | 10 | 0.0131 | 0.0199 | 0.8939 |
|  |  |  | 20 | 0.0225 | 0.0291 | 0.9023 |
|  |  |  | 30 | 0.0290 | 0.0348 | 0.9092 |
|  |  |  | 50 | 0.0379 | 0.0425 | 0.9192 |
|  | 0.5/0.9 | 1.0000 | 5 | 0.0099 | 0.0167 | 0.8900 |
|  |  |  | 10 | 0.0208 | 0.0278 | 0.8970 |
|  |  |  | 20 | 0.0358 | 0.0416 | 0.9077 |
|  |  |  | 30 | 0.0457 | 0.0504 | 0.9155 |
|  |  |  | 50 | 0.0583 | 0.0618 | 0.9265 |

Table S5 Table of notations in the article

| Symbol | Definition | Connection |
| --- | --- | --- |
| *N* | The number of historical trials |  |
| $n_{i}$ | The sample size of the *i*-th trial |  |
| PPV | Positive predictive value |  |
| NPV | Negative predictive value |  |
| $D_{pi}$ | The biomarker response identifier of the *p*-th patient in the *i*-th trial |  |
| $C_{pi}$ | The clinical response identifier of the *p*-th patient in the *i*-th trial |  |
| $B_{Ti}$ | The number of biomarker response in the treatment group of the *i*-th trial | $B_{Ti}=\sum_{p=1}^{n_{i}/2} (D_{pi}=1\vert\text{treatment group})$ |
| $B_{Ci}$ | The number of biomarker response in the control group of the *i*-th trial | $B_{Ci}=\sum_{p=1}^{n_{i}/2} (D_{pi}=1\vert\text{control group})$ |
| $B_{i}$ | The number of biomarker response in the *i*-th trial | $B_{i}=B_{Ti}+B_{Ci}=\sum_{p=1}^{n_{i}} (D_{pi}=1)$ |
| $X_{Ti}$ | The number of clinical response in the treatment group of the *i*-th trial | $X_{Ti}=\sum_{p=1}^{n_{i}/2} (C_{pi}=1\vert\text{treatment group})$ |
| $X_{Ci}$ | The number of clinical response in the control group of the *i*-th trial | $X_{Ci}=\sum_{p=1}^{n_{i}/2} (C_{pi}=1\vert\text{control group})$ |
| $X_{i}$ | The number of clinical response in the *i*-th trial | $X_{i}=X_{Ti}+X_{Ci}=\sum_{p=1}^{n_{i}} (C_{pi}=1)$ |
| $\pi_{BTi}$ | The biomarker response rate of the treatment group in the *i*-th trial |  |
| $\pi_{BCi}$ | The biomarker response rate of the control group in the *i*-th trial |  |
| $\pi_{XTj}$ | The clinical response rate of the treatment group in the *j*-th trial | $\pi_{XTj}=\pi_{BTj}\times\text{PPV+}(1-\pi_{BTj})\times(1-\text{NPV})$ |
| $\pi_{XCj}$ | The clinical response rate of the control group in the *j*-th trial | $\pi_{XCj}=\pi_{BCj}\times\text{PPV+}(1-\pi_{BCj})\times(1-\text{NPV})$ |
|  | The proportion of biomarker responses in the treatment group in the *i*-th trial when equal sample size in the two groups. | . It is estimated by for equal sample size and for unequal sample size. $R$ is the sample size ratio of treatment and control group. |
|  | The proportion of clinical responses in the treatment group in the *i*-th trial when equal sample size in the two groups. | It is estimated by for equal sample size and for unequal sample size. |
| $RR_{j}$ | The rate ratio on clinical response of the *j*-th trial |  |
| *VE* | Vaccine efficacy | $VE=1-RR$ |
